# Comparison and Intercorrelation of Various Bentonite Products for Oenological Properties, Elemental Compositions, Volatile Compounds and Organoleptic Attributes of White Wine

**DOI:** 10.3390/foods12020355

**Published:** 2023-01-11

**Authors:** Nándor Rakonczás, Zoltán Kállai, Béla Kovács, Gabriella Antal, Szilárd Szabó, Imre J. Holb

**Affiliations:** 1 Institute of Horticulture, University of Debrecen, Böszörményi út 138, 4032 Debrecen, Hungary; 2 Department of Genetics and Applied Microbiology, University of Debrecen, Egyetem tér 1, 4032 Debrecen, Hungary; 3 Institute of Food Science, University of Debrecen, Böszörményi út 138, 4032 Debrecen, Hungary; 4 Department of Physical Geography and Geoinformatics, University of Debrecen, Egyetem tér 1, 4032 Debrecen, Hungary; 5 Eötvös Loránd Research Network (ELKH), Centre for Agricultural Research, Plant Protection Institute, 1022 Budapest, Hungary

**Keywords:** bentonite fining, macro- meso- micro-elements, acids, sensory parameters, aroma compounds, Pearson correlation, factor loadings, biplot

## Abstract

Bentonite fining is one of the generally applied wine-making technological elements that may seriously affect wine components. The aim of this study was (i) to investigate the effect of 21 bentonite products on eight oenological parameters, 19 elements, 21 volatile organic compounds (VOCs) and 10 organoleptic properties of white wine; and (ii) to quantify intercorrelations among the parameters separately for each of the four quality attributes. Among oenological parameters, sugar, acidity, malic-, lactic-, citric acid and total phenol contents were significant among several bentonite products. The amounts of elements were the lowest in the control wine treatments (with exceptions of, e.g., Ni and Cu); and these values were significantly different from several bentonite products. The relative presence of the VOCs was above 100% for most VOCs, but it was below 100% for 1-propanol, 4-amino-1,5-pentandioic acid and butane-dioic acid, and diethyl ester in all treatments. For organoleptic parameters, the values of clearness, colour, flavour intensity and taste persistency was the lowest in the control wine treatment, while the values of flavour character, flavour quality, taste intensity, taste character, and overall harmony were the highest for the bentonite products of AP, EBE, M-SA, EBE, EBE, respectively. Results of correlation and factor analyses showed strong intercorrelative effects of bentonite fining on the four quality attributes. In conclusion, this study can help in the proper choice of a specific bentonite product in relation to complexity effects of bentonite fining.

## 1. Introduction

Wine contains numerous compounds, including polyphenols, elemental composition, and volatile organic compounds (VOCs) (e.g., [1]). Several studies have shown that moderate wine consumption has several positive effects on human health due to, e.g., phenolic compounds [2,3,4]. These compounds in wine can protect human health via anti-microbial, anticancer, cardioprotective, hepatoprotective and neuroprotective activities. The phenolic compounds of wine also have a key effect on the oenological parameters of the wine, but the sensory properties (e.g., colour, astringency and bitterness) are also directly influenced by the phenolic composition of the wine (e.g., [5,6,7,8]). The various chemical constituents of wine are among those most investigated features. Elemental composition directly influences the qualitative characteristics of the wine, such as total acidity, alcohol, dry extract and residual sugar [9]. Metals among elemental compositions also affect the organoleptic characteristics of wine [10]. In addition, the amount and type of VOCs may be affected by several factors, such as geographical origin, aging, alcoholic and acetic fermentation as well as technological processes [11,12]. The characterization of the volatile fraction helps to improve the quality of the wine product [12].

The bentonite fining of wine has been generally applied in the technology for a long time in order to eliminate thermolabile proteins, which cause haziness in wine [13]. These proteins are positively charged and easily react with the negatively charged particles of bentonite (mainly montmorillonite: Al_2_O_3_-4SiO_2_-H_2_O). This results in flocculation, followed by sedimentation. In this process, not only are the thermolabile proteins eliminated, but the mineral background and phenolic and volatile constituents also change. This process can be bentonite product-specific and may influence the terroir-character of the wine. Bentonite is one of the most efficient fining agents for reaching protein stability in wine; however, bentonite may influence seriously the quality of wine [14].

Among oenological parameters, previous studies showed various results on bentonite fining. For example, titratable acidity, ethanol, and tartaric acid contents were not different between the bentonite and control treatments. However, malic acid contents were significantly lower in the bentonite treatment compared to the control treatment during wine bottling [15], and in the study of Maslov-Bandic et al. [16] the sugar content was also significantly lower in the bentonite treatments compared to the control.

Previous studies showed that elemental compositions of wine were the most affected parameters during bentonite finings. In some cases, bentonite fining increased the amounts of some micro-elements in the wine, such as Na, Al and Ca [10,17,18,19,20] and Fe, Sr and Ba [21]. However in other cases, previous wine studies demonstrated that Cu, K and Zn contents [22] and also B content [23] decreased in the bentonite treatments compared to the control. The largest decrease was achieved for Cu (−43%) [22]. Previous results also showed that different bentonite products may cause various changes in the elemental composition of the wine, as bentonite quality (clearness, activity, absorption capacity and the surface of the particles) is dependent upon the place of origin [23,24,25]. As a consequence, bentonite fining not only influences the elemental composition of the wine but is a main source of mineral contamination too [22,23,26,27].

In the case of the VOCs, Horvat et al. [14] demonstrated that bentonite affected wine quality by changing some key fermentation volatiles compared to treatments without bentonite fining. Bentonite fining may reduce aroma and flavour compounds in the wine due to direct adsorption and deproteinization [28,29,30,31,32,33]. The VOCs loss was verified after multiple treatments in various wine types [32,34]. The results of Sanborn et al. [35] indicated that fining agents can perform unpredictably and may result in various levels of wine quality reductions. Several studies showed that the loss of VOCs in wine after bentonite treatments severely affects the sensory attributes of the wine too [28,29,32,33,34].

Overall, previous studies verified that bentonite fining affects many wine parameters including oenological, elemental, volatile and sensory attributes, but most studies measured only parts of the parameters and only few bentonite products; and therefore, an overall evaluation may help to better characterize the effect of a given bentonite product on wine quality.

Correlations among several wine attributes (including elemental, volatile, and sensory parameters) in bentonite treatments were investigated [16,21,36,37,38]. However, intercorrelation among the parameters were not shown in an overall evaluation with different wine attributes and large numbers of bentonite products, which may give a better understanding of the relationship between the bentonite fining process and wine quality changes.

The aim of this study was firstly to investigate the effect of twenty-one bentonite products together with three control treatments on eight oenological parameters, nineteen elements, twenty-two VOCs and ten organoleptic properties of white wine that originated from the Eastern Hungarian wine regions (Debrecen, Hajdú-Bihar county); and secondly to quantify intercorrelations (Pearson correlation, factor analyses) among the parameters separately for oenological, elemental, volatile, and organoleptic properties in order to highlight the best relationships of the correlated measures.

## 2. Materials and Methods

### 2.1. Experimental Design: Wine Material, Bentonite Products and Treatments

The examined wine was made from varieties of ‘Bianca’ (70%), ‘Furmint’ plus ‘Harslevelű’ (30%). The plantation was established in 1998 in the Experimental Horticultural Station, University, Debrecen-Pallag (47°35′20”/21°38′23”; 128 m above sea level). Harvest was carried out in late September, 2017. The wine for the experiment was prepared from the three varieties as follows. The harvested grape was cooled down to 12 °C before processing, then 100 mg/kg potassium metabisulphite was sprinkled into the grapes during the processes of crushing and destemming. Pressing started immediately after destemming, and 2 g/10 L yeast was added a day later (Mycoferm A-R-T, Interker-Wein, Eger, Hungary). Decantation was not applied because the grapes were clean and healthy. After the start of the fermentation process at 20 °C, the wine must in 11 × 120-litre plastic casks was transported to a cooling chamber at 12 °C. The fermentation process ended after three weeks. At the end, to support the completion of sugar degradation, the plastic casks were placed at 15 °C. Within a week, the wine was racked open with the joint application of sulfitization (50 mg/L, with SO_2_ solution, Interker-Wein, Eger, Hungary), and the casks were filled up with minimal air lock. The base wine was put together into an IBC plastic container in the middle of December (2017) with open racking and the addition of 30 mg/L supplementary sulfitization. Fining was performed in spring 2018 and then bentonite fining treatments were performed.

To perform the treatments, 20 L of wine samples were racked into 30-litre plastic drums, which were fined with medium doses of 18 bentonite products according to producers’ recommendations (Table 1). The experimental design also included three control treatments (C, SC, SMC) in order to represent the complexity of the procedure (Table 1). C treatment contains only the wine sample and did not get any additional amendments. SC treatment contains the wine sample with 10 mg/L sulfitization. Sulfitization was applied to counterbalance the oxidation in the wine. In the SMC treatment, apart from the sulfitization a mixing was applied with the same speed and timing as in the bentonite product treatments (full rotation with 30 s). Assays were carried out in triplicate.

Preparation of bentonite treatments was as follows: each bentonite product was previously hydrated according to the producers’ instructions. Prepared products were mixed into the wine with a drill mixer for 30 s with full rotation besides intensive aeration. After the collapse of the foam (some minutes), the solutions were racked into new glass jugs previously rinsed with hot tap water (measure of capacity: 15 L). Then 10 mg/L sulfitization was applied at racking. Jugs were closed with plastic caps with no headspace over the wine and were put into a cool chamber set to 12 °C. According to the concept of the trial, wine was left over the bentonite sediment. The wine samples were taken two weeks after bentonite treatments. Dissolved oxygen was measured with a HI 9146 equipment (HANNA Instruments Service Ltd., Szeged, Hungary) before bottling of the wine samples (Table 1).

### 2.2. Mesurements

All measurements were prepared according to the European Commission Regulation (EEC) No. 2676/90.

#### 2.2.1. Oenological Parameters

Eight oenological parameters (ethyl-alcohol, sugar, total acidity, tartaric acid, malic acid, lactic acid, citric acid, volatile acid and total phenol) of the white wine sample of the twenty-one treatments were determined according to the official standards of the International Organisation of Vine and Wine [33]. Parameters were measured in three replicates. Ethyl-alcohol content was expressed as *v*/*v*%. Sugar, total acidity, tartaric acid, malic acid, lactic acid, citric acid, and volatile acid contents was given as g/L and total phenol contents as mg/L.

#### 2.2.2. Elemental Composition

Quantities of eight macro- and meso elements (P, K, Ca, Mg, S, Al, Na, and B) and eleven micro elements (Cr, Mn, Fe, Co, Ni, Cu, Zn, Sr, Cd, Ba, and Pb) were determined and expressed in mg/L and µg/L, respectively, from the white wine samples treated with twenty-one bentonite products, including three control treatments. Elemental composition was determined with ICP OES and ICP MS, similarly to the study of Rakonczás et al. [21]. Briefly the used determination method was the following. Before the analysis, wine samples were diluted ten-fold with 5 (m/V) % nitric acid (VWR International Ltd., Debrecen, Hungary). A deionized water type-1 grade was used by using a Milli-Q^®^ water purification system (Merck-Millipore, Molsheim, France). Three replicates were analyzed from each sample. The elemental analysis was measured by an iCAP 6300 ICP-OES instrument equipped with a CETAC ASX-520 autosampler and by a Meinhard-type concentric nebulizer attached to a cyclonic spray chamber (Thermo Fischer Scientific, Cambridge, UK). All measurements were made in axial-mode. An external calibration was made for the element quantification. A multi-element solution was made from mono-element standards using a 1000 mg/L solution in 2% nitric acid (Scharlab, Sentmenat, Spain). This solution was used as a stock solution and used to construct calibration curves for appropriate dilutions. Each sample was measured in three replicates.

#### 2.2.3. Volatile Organic Compounds (VOCs)

Twenty-two VOCs were determined from the white wine samples in the twenty-one bentonite product treatments, including three control treatments (Table 2). The profile of the VOCs in each wine sample was determined by a Bruker Scion 456-gas chromatograph equipped with a Bruker SHS-40 Headspace Sampler (Bruker Corporation, Billerica, MA, USA). The equipment was supplemented with a Bruker SQ mass spectrometer and with a Br-5 capillary column (30 m 0.25 mm i. d. 1.0 µm film thickness).

Helium was used as a carrier gas, applied in a constant flow mode with a flow rate of 1 mL/min. In the headspace vials, 5000 µL solution samples were applied at 60 °C for 20 min. in the automatic sampler with added sodium chloride (1 g) and with no agitation, and then 1000 µL headspace sample was injected into the column. The transfer line was maintained at 230 °C, and the injector temperature was 250 °C (20:1 split time ratio). The initial temperature of the oven was 40 °C, held for 2 min. Then the temperature was increased to 280 °C at 10 °C min^−1^, and this temperature was held for 3 min. Electron impact ionisation mode (70 eV) was applied for the mass spectrometer with a source temperature of 180 °C, with a scanning rate of 1 scan per second, and with a full scan mode. Mass spectrometric data were used for the identification of the VOCs (National Institute of Standards and Technology (NIST), version 2005, mass spectral library). A semi-quantitative analysis was performed to identify VOCs. In the analysis, changes in volatile compounds caused by bentonite products were more essential for our study than the exact concentration of volatile compounds. Therefore, the relative presence of VOCs is expressed in the percentage of control (C) treatment.

#### 2.2.4. Organoleptic Evaluation

The organoleptic evaluation of the wine samples in the twenty-one bentonite product treatments was performed according to OIV 332A/2009 resolution. The procedure of organoleptic evaluation of the wine samples was made by a panel of 14 trained students between the ages of 20 and 24 (University Debrecen, Hungary) according to Jackson [39] and Hopfer & Heymann [40]. The panel members took part in a one-semester theoretical and practical course including 3 h theory and 3 × 4 h thematic wine-tasting occasions: white wine, red wine, technological wines and wines of the world. The environmental, material and personal conditions during the sensory evaluation were created taking into account the MSZ 9462-81 standard. Written consent and ethics approval were arranged for the organoleptic assessments. All evaluations were done independently and in three replicates. Each panel member was asked to evaluate clearness, colour, flavour intensity, flavour character, flavour quality, taste intensity, taste character, taste quality, taste persistency, and overall harmony. The assessment guide, containing a scale for each organoleptic parameter, including the two anchors, was as follows: skin and flesh colour: 0 = unacceptable, 9 = excellent; flavour intensity and character: 0 = very weak, 8 = very strong; flavour quality: 0 = unacceptable and 15 = excellent; taste intensity, character and persistency: 0 = very bad, 8 = excellent; taste quality: 0 = very bad, 20 = excellent and overall harmony: 0 = unacceptable, 10 = excellent.

### 2.3. Data Analyses

#### 2.3.1. ANOVA

For oenological, elemental, volatile, and organoleptic measure types, a randomized complete block design (RCBD) was used to design the bentonite treatments. ANOVA was performed to analyze the data set of all measurements in the four measurement types using an SPSS 19 program (SPSS Inc., Chicago, IL, USA). The effects of twenty-one bentonite product treatments including the three control treatments were evaluated on all measurements of the oenological, elemental, volatile, and organoleptic measure types. The LSD t-test was used to separate treatment means at *p* = 0.05 levels.

#### 2.3.2. Pearson Correlation Analyses

The relationship among the measurements was quantified separately for oenological, elemental, volatile, and organoleptic measure types. In order to quantify relationships among the measurements, Pearson’s correlation coefficients were determined for the relationships of the measures of the four measure types in all combinations (55, 154, 214, and 46 variable pairs for oenological, elemental, volatile, and organoleptic measure types, respectively. Pearson’s correlation analyses were done by using Genstat 5 Release 4.1 (Lawes Agricultural Trust, IACR, Rothamsted, UK).

#### 2.3.3. Principal Axis Factor Analyses with Varimax Rotation

Factor analysis using Genstat 5 Release 4.1 (Lawes Agricultural Trust, IACR, Rothamsted, UK) was done separately for oenological, elemental, volatile, and organoleptic measure types. The aim of factor analyses was to select those measurements that can characterize the best white wine in the bentonite product treatments. In a factor analysis model, each measurement is represented as a linear equation of *n* hypothetical factors, *f_1_*, *f*_2_, *f*_3_, … *f_n_*:

*M_i_* = *a*_*i*1_*f*_1_ + *a*_*i*2_*f*_2_ + *a*_*i*3_*f*_3_ and + … + *a_ij_f_j_* + *R_i_*,

where *M_i_* is the *i*th measurement (e.g., *M*_1_ = ethyl-alcohol content); *f_j_* is the *j*th factor; *a_ij_* is the factor loading representing the correlation of the measurement *i* with factor *j*; and *R_i_* represents a residual component not accounted for by the factors. A principal axis procedure was used to calculate factor loadings followed by a Varimax rotation according to the study of Kaiser [41]. Four factors were presented and significant factor loadings were highlighted separately for the four measure types. Biplot diagrams were also prepared to visualise the first two factors (Factor 1 vs. Factor 2) with the distributions of the measure types and bentonite products. The biplot diagrams were performed separately for oenological, elemental, volatile, and organoleptic measure types with the statistical package of R 1.3.30 [42] with the MultBiplotR [43].

## 3. Results

### 3.1. Oenological Parameters

Oenological parameters characterized a dry white wine (mean residual sugar content: 0.57 g/L) with a mild, intermediate acidity (mean titratable acid content: 6.11 g/L) and considerably high ethyl-alcohol content (mean ethyl-alcohol content: 12.05 *v/v*%) (Table 3). Among the eight oenological parameters, ethyl-alcohol, tartaric acid and volatile acid contents were non-significant among the bentonite product treatments including control treatments (Table 3). The lowest sugar content of the sampled wine (0.4 g/L) was measured in NCPE and the highest (1.2 g/L) in SC (Table 3). The sugar content of the sampled wine was 0.5 and 0.6 g/L for the bentonite products with the exceptions of EBS, NCPE, and EG products (Table 3). The lowest titratable acidity was measured in the C treatment (5.6 g/L) and the highest (6.2 g/L) in SMC treatment. The lowest total phenol was measured in the SC treatment (10.2 mg/L) and the highest (21.0 mg/L) in C. Values in the treatments of bentonite products ranged between 6.01 and 6.19 g/L for titratable acidity and between 16.4 and 18.0 mg/L for total phenol (Table 3). The largest values were 2.1 g/L in SC treatment for malic acid, 1.6 g/L in C treatments for lactic acid and 0.49 g/L in SC treatment for citric acid.

### 3.2. Chemical Elements

#### 3.2.1. Macro- and Meso Elements

The amounts of measured macro- and meso elements in the sampled wine were the lowest in the control (C) treatments (Table 4).

P contents of the sampled wine were 66.7, 74.9 and 73.6 mg/L in the C, SC, and SMC treatments, respectively, and ranged between 70.1 and 75.9 mg/L in the eighteen bentonite product treatments (Table 4). The P content in all bentonite product treatments was significantly higher than in the control (C) treatment, with the exception of P and GPT treatments.

K contents of the sampled wine were 432.7, 470.5 and 479.3 mg/L in the C, SC, and SMC treatments, respectively, and ranged between 466.3 and 496.2 mg/L in the eighteen bentonite product treatments (Table 4). The K content in all bentonite product treatments was significantly higher than in the control (C) treatment.

Ca contents of the sampled wine ranged between 41.22, 41.87 and 45.81 mg/L in the C, SC, and SMC treatments, respectively, and three values were not significantly different from each other (Table 4). The Ca values of the eighteen bentonite product treatments ranged between 47.02 and 53.3 mg/L (Table 4), and all bentonite product treatments were significantly higher than in the control (C) and sulfur content (SC) treatments.

Mg content of the sampled wine ranged between 41.8 and 47.3 mg/L in the twenty-one treatments including the three control treatments (Table 4). Mg values of all eighteen bentonite treatments were significantly different from Mg values of the control (C) treatment (Table 4).

S content of the sampled wine was the lowest in the C treatment (5.845 mg/L) and the highest in the NCPE treatment (9.181 mg/L); and the highest value was significantly different from S values of all treatments, with the only exceptions being AP and EBE treatments. S content ranged between 5.845 and 6.955 mg/L in the three control treatments (C, SC, and SMC), and the C values were significantly different from the SC treatment (Table 4). The S values of the eighteen bentonite product treatments ranged between 6.091 and 9.181 mg/L (Table 4), and S values of most bentonite product treatments were significantly higher than in the control (C) except for FB, EC, EB, B, GTP, P and EG treatments.

Al and Na contents of the sampled wine were 0.27, 0.296 and 0.302 mg/L, and 7.75, 8.82 and 8.63 mg/L, respectively (Table 4). Al contents in the three control treatments were significantly different from the eighteen bentonite product treatments with the exceptions of M-SA, CR, EB, P and EG treatments. Na contents in the FB, BW, GTP and EBS treatments were significantly higher than the Na content values in the three control treatments, and the Na content values of the FB, BW and GTP treatments were at least twice higher compared to the three control treatments (Table 4).

B content of the sampled wine ranged between 0.526 and 0.603 mg/L in the twenty-one treatments (Table 4). The B content in the M-SA treatment (0.603 mg/L) was significantly different only from the control (C) treatment (0.526 mg/L), and all other treatments were non-significant from each other (Table 4).

#### 3.2.2. Micro Elements

Cr, Fe and Co contents of the sampled wine were 1.58, 1.71 and 1.63 µg/L, 1047, 1111 and 1105 µg/L, and 1.56, 1.69 and 1.72 µg/L in the C, SC, and SMC treatments, respectively, (Table 5). Cr and Co contents (2.85 and 2.86 µg/L, respectively) were the highest in the FB treatment while the Fe content (2132 µg/L) was the highest in the BW treatment. The highest Cr content in the FB treatment was significantly higher than all other treatments, with the exception of the BW treatment. The highest Co content in the FB treatment was significantly higher than all other treatments with the exceptions of the BW and EO treatments. The highest Fe content in the BW treatment was significantly higher than all other treatment (Table 5).

Mn contents of the sampled wine were 725.7, 747.4 and 741.2 µg/L in the C, SC, and SMC treatments, respectively, and ranged between 718.5 and 886.2 µg/L in the eighteen bentonite product treatments (Table 5). The Mn content was the highest in the AP treatment, which was significantly higher than all other treatments with the exceptions of the NCPE, GPT, and EBE treatments.

Ni contents were the lowest in the C and SMC treatments (12.5 and 13.7 µg/L, Table 5). The highest Ni value was in the FB treatment (17.9 µg/L); however, this value was not significantly different from those of several treatments (M-SA, NCPE, CR, BW, B, GPT, EO, BCD, and BF).

The Cu contents of the sampled wine ranged widely in the twenty-one treatments (values ranged from 6.42 to 23.2 µg/L, Table 5). Cu content was the lowest (6.42 µg/L) in the GTP treatment, and this value was significantly lower than in all other treatments. The highest Cu content (23.2 µg/L) was in the sulfur control (SC) treatment, and this value was significantly higher than in all other treatments (Table 5).

The Zn contents of the sampled wine ranged between 209.5 and 263.5 µg/L (Table 5). The Zn content was the lowest (209.5 µg/L) in the control (C) treatment, and this value did not significantly differ from values in the EC, BW, P and EG treatments. The highest Cu content (263.5 µg/L) was in the M-SA treatment, and this value was significantly higher than in the C, EC, BW, P, and EG treatments (Table 5).

The Sr contents of the sampled wine ranged between 316.2 and 431.3 µg/L (Table 5). The Sr content was the lowest (316.2 µg/L) in the control (C) treatment, and this value did not significantly differ from values in the SC, SMC, M-SA, EB, P, and EG treatments. The highest Sr content (431.3 µg/L) was in the NCPE treatment, and this value was significantly higher compared to values of all treatments except for the treatments of GTP and BCD (Table 5).

Among the micro-elements, Cd contents of the sampled wine was the lowest (between 0.198 and 0.413 µg/L) (Table 5). The Cd content was the lowest (0.198 µg/L) in the control (C) treatment, and this value did not significantly differ from values in the SC, SMC, EBS, M-SA, EC, EBS, FB, EC, EB, AP, N, EO, P, and EG treatments. The highest Cd content (0.413 µg/L) was in the CR treatment, and this value was significantly higher compared to values of all treatments except for the treatments of M-SA, NCPE, BW, B, GTP and BCD (Table 5).

Ba contents of the sampled wine ranged between 39.67 and 81.95 µg/L (Table 5). The Ba content was the lowest (39.67 µg/L) in the control (C) treatment, and this value did not significantly differ from values in the SC, M-SA, P, and EG treatments. The highest Ba content (81.95 µg/L) was in the CR treatment, and this value was significantly higher than the values of all treatments (Table 5).

Pb contents of the sampled wine ranged between 2.27 and 9.46 µg/L (Table 5). The Pb content was the lowest (2.27 µg/L) in the control (C) treatment, and this value did not significantly differ from values in the SC, SMC, M-SA, EB, and EG treatments. The highest Pb content (9.46 µg/L) was in the CR treatment, and this value was significantly higher than the values of all treatments (Table 5).

### 3.3. Volatile Organic Compounds (VOCs)

The relative presence of the twenty-two VOCs, expressed in the percentage of control (C) treatment, ranged from 18.5 to 169.8% (Table 6). The lowest percentage (18.5%) was detected for the VOCs of phenol, 2-methoxy- in the EO treatment, while the highest (169.8%) was for acetic acid in the CR treatment. The VOCs of ‘phenol, 2-methyl-‘, ‘butanedioic acid, diethyl ester’, ‘δ-dodecalactone’, ‘1-propanol, 2-methyl-‘, and ‘octanoic acid, ethyl ester’ were not detected in seven (FB, M-SA, NCPE, BW, EB, EO, BF) five (FB, EB, N, EO, P), three (FB, EO, EG), one (GPT), and one (EO) treatments, respectively. The lowest number of VOCs (18) were detected in the EO treatment (Table 6).

The relative presence of the VOCs was below 100% for ‘1-propanol’, ‘4-amino-1,5-pentandioic acid’ and ‘butanedioic acid, diethyl ester’ in all bentonite treatments (Table 6). The relative presence of the VOCs was above 100% for ‘acetic acid’, ‘ethyl acetate’, ‘1-propanol, 2-methyl-‘, ‘δ-dodecalactone’, ‘propanoic acid, ethyl ester’, ‘1,3-dioxolane, 2,4,5-trimethyl-‘, ‘1-butanol, 3-methyl-‘, ‘1-butanol, 2-methyl-, (S)-‘, ‘propanoic acid, 2-methyl-, ethyl ester’, ‘isobutyl acetate’, ‘triethyl borate’, ‘butanoic acid, ethyl ester’, ‘1-hexanol’, ‘1-butanol, 3-methyl-, acetate’, ‘hexanoic acid, ethyl ester’, ‘phenol, 2-methyl-‘, ‘phenol, 2-methoxy-‘, ‘phenylethyl alcohol’ and ‘octanoic acid, ethyl ester’ in five (SMC, CR, BW, AP, EBE), five (SC, SMC, N, GPT, BCD), four (SC, SMC, NCPE, BCD), four (NCPE, CR, AP, N), two (NCPE, GPT), one (GPT), four (SC, SMC, NCPE, BCD), four (SC, SMC, NCPE, BCD), one (GPT), four (SC, SMC, NCPE, N, BCD), six (NCPE, EC, BW, AP, BCD, EBE), four (SC, SMC, N, GPT), one (SC), three (SMC, N, GPT), two (NCPE, N), three (SC, SMC, BCD), four (SC, EC, GPT, BCD), four (SC, EBS, BW), and three (SMC, NCPE, GPT) treatments, respectively. The highest numbers of VOCs (10), above 100% relative presence, were detected in the SMC treatment (Table 6).

The relative presence of the VOCs of ‘1-propanol’ in the control (C) treatment was significantly higher than the values in the FB, B, GPT, EO, P, and BF treatments (Table 6). The relative presence of the VOCs of ‘acetic acid’ in the C treatment was significantly lower than the values in the CR and AP treatments. The relative presence of the VOCs of ‘ethyl acetate’ in the C treatment was significantly higher than the values in the FB, EB, B, EO, and BF treatments. The relative presence of the VOCs of ‘1-propanol, 2-methyl-‘ in the C treatment was significantly higher than the values in the FB, M-SA, CR, EB, B, EO, P and BF treatments. The relative presence of the VOCs of ‘δ-dodecalactone’ in the C treatment was significantly lower than the values in the NCPE treatment. The relative presence of the VOCs of ‘propanoic acid, ethyl ester’ in the C treatment was significantly higher than the values in the FB, M-SA, EC, BW, CR, EB, B, EO, P, EG and BF treatments. The relative presence of the VOCs of ‘1,3-dioxolane, 2,4,5-trimethyl-‘ in the C treatment was not significantly different from the values of the SC, SMC, NCPE, N, GPT, and BCD treatments. The relative presence of the VOCs of ‘1-butanol, 3-methyl-‘ in the C treatment was significantly higher than in the FB, M-SA, EB, B, EO, P, EG, and BF treatments. The relative presence of the VOCs of ‘1-butanol, 2-methyl-, (S)-‘ in the C treatment was significantly lower than in the SC, SMC, NCPE and BCD treatments. The relative presence of the VOCs of ‘4-amino-1,5-pentandioic acid’ in the C treatment was not different significantly from in the SC, SMC, N, GPT, EO and BCD treatments. The relative presence of the VOCs of ‘propanoic acid, 2-methyl-, ethyl ester’ in the C treatment was significantly higher than in the FB, EC, CR, EB, AP, B, EO, P, EG, and BF treatments (Table 6).

The relative presence of the VOCs of ‘isobutyl acetate’ in the C treatment was significantly higher than in the FB, BW, and EO treatments (Table 6). The relative presence of the VOCs of ‘triethyl borate’ in the C treatment was significantly higher than in the SC, FB, EB, B, EO, P, EG and BF treatments. The relative presence of the VOCs of ‘butanoic acid, ethyl ester’ in the C treatment was significantly higher than in the FB, M-SA, EB, EO and BF treatments. The relative presence of the VOCs of ‘1-hexanol’ in the C treatment was not different significantly from that in the SC, SMC, NCPE, BW, AP, N, GPT, BCD, and EBE treatments. The relative presence of the VOCs of ‘1-butanol, 3-methyl-, acetate’ in the C treatment was significantly higher than in the EB, B, EO, EG and BF treatments. The relative presence of the VOCs of ‘hexanoic acid, ethyl ester’ in the C treatment was significantly higher than in the FB, M-SA, EB, AP, EO, P, EG and BF treatments. The relative presence of the VOCs of ‘phenol, 2-methyl-‘ in the C treatment was significantly higher than in the EC, CR, AP, B, N, P and EG treatments. The relative presence of the VOCs of ‘phenol, 2-methoxy-‘ in the C treatment was significantly higher than in the EBS, FB, M-SA, NCPE, CR, BW, EB, N, EO, P and BF treatments. The relative presence of the VOCs of ‘phenylethyl alcohol’ in the C treatment was not significantly different from that in the SC, SMC, EBS, BW, N, P and EBE treatments. The relative presence of the VOCs of ‘butane-dioic acid, diethyl ester’ in the C treatment was significantly higher than that in the M-SA, NCPE, EC, CR, AP, B, GPT, EG and BF treatments. The relative presence of the VOCs of ‘octanoic acid, ethyl ester’ in the C treatment was significantly higher than in FB, EC, EB, B, EG and BF treatments (Table 6).

### 3.4. Organoleptic Parameters

Clearness indices of the sampled wine ranged between 3.23 and 4.62; the lowest value was obtained in the CR and FB treatments and the highest in the EBE treatment (Table 7). The lowest clearness index was not significantly different from the values of the SC, M-SA, NCPE and N treatments. However, the highest clearness index was significantly higher than in all other treatment.

Colour indices of the sampled wine ranged between 7.54 and 8.62; the lowest values were obtained in the SC, FB, CR and N treatments and the highest in the EBE treatment (Table 7). The lowest colour index was significantly different from values of BW, GPT and EBE treatments.

Flavor intensity indices of the sampled wine ranged between 4.77 and 6.15; the lowest value was obtained in the M-SA treatment and the highest in the BF treatment (Table 7). The lowest flavour intensity index was not significantly different from values of SMC and EC treatments. The highest flavour intensity index was significantly higher than in all other treatments with the exceptions of the SC, EBS, BW, EB, AP and EO treatments.

Flavor character indices of the sampled wine ranged between 3.54 and 4.52; and the lowest value was obtained in the EBE treatment and the highest one in the AP treatment (Table 7). The lowest flavour character index was not significantly different from values of SMC, M-SA, EC, B, N, BCD and EBE treatments. The highest flavour character index was significantly higher than in all other treatments with the exceptions of the C, SC, BW and BF treatments.

Flavor quality indices of the sampled wine ranged between 10.62 and 12.46; the lowest value was obtained in the EBE treatment and the highest in the EB and AP treatments (Table 7). The lowest flavour quality index was not significantly different from the values of the FB and M-SA treatments. The highest flavour quality index was significantly higher than the values of the FB, M-SA, EC, EG and EBE treatments.

Taste intensity indices of the sampled wine ranged between 5.62 and 6.46; the lowest value was obtained in the M-SA treatment and the highest in the SC treatment (Table 7). The lowest taste intensity index was significantly lower than the values of the SC, SMC, CR, EB, AP, B, GPT, EO, BCD and BF treatments. The highest taste intensity index was significantly higher than the values of the FB, M-SA, NCPE, N and EBE treatments.

Taste character indices of the sampled wine ranged between 3.77 and 5.77; the lowest value was obtained in the EBE treatment and the highest in the SC treatment (Table 7). The lowest taste intensity index was not significantly different from the values of the C, EBS, FB, M-SA, NCPE, EC, N, EO, P, BCD and EG treatments. The highest taste character index was significantly higher than the values of all other treatments.

Taste quality indices of the sampled wine ranged between 14.62 and 16.92; the lowest value was obtained in the FB treatment and the highest in the SMC and BF treatments (Table 7). The lowest taste intensity index was not significantly lower than the values of the SC, EBS, M-SA, NCPE, EC, CR, N, P, BCD and EBE treatments. The highest taste quality index was not significantly higher than the values of the EB, AP, B, EO and EG treatments.

Taste persistency indices of the sampled wine ranged between 5.62 and 6.23; the lowest values were obtained in the C, FB, M-SA, and NCPE treatments and the highest in the SC treatment (Table 7). The lowest taste persistency index was significantly lower than the values of the SC, SMC, EB and AP treatments. The highest taste persistency index was significantly higher than the values of the C, FB, M-SA, and NCPE treatments.

Overall harmony indices of the sampled wine ranged between 8.46 and 9.54; the lowest value was obtained in the EBE treatment and the highest in the AP treatment (Table 7). The lowest overall harmony index was significantly lower than the values of the SMC, EB, AP and BF treatments. The highest overall harmony index was significantly higher than the values of the FB, M-SA, EC and P treatments.

### 3.5. Relationship among Parameters

#### 3.5.1. Pearson Correlation and Factor Analyses for Oenological Parameters

When the oenological parameters data set were analyzed for all bentonite products together, 55 parameter pairs were correlated. Of the 55 pairs, 18 correlated significantly at *p* = 0.05 probability level (Table 8). Among these 18 pair-variables, nine were correlated positively (alcohol versus (vs.) tartaric acid, sugar vs. citric acid, acidity vs. malic acid, acidity vs. citric acid, acidity vs. sulphur-bound, malic acid vs. citric acid, malic acid vs. sulphur-bound, citric acid vs. sulphur-bound and volatile acid vs. total phenol) and nine negatively (sugar vs. volatile acid, sugar vs. total phenol, acidity vs. lactic acid, acidity vs. volatile acid, malic acid vs. volatile acid, malic acid vs. total phenol, citric acid vs. volatile acid, citric acid vs. total phenol and volatile acid vs. sulphur-bound) indicating a strong connection among basic parameters (Table 8).

**Figure 1:**
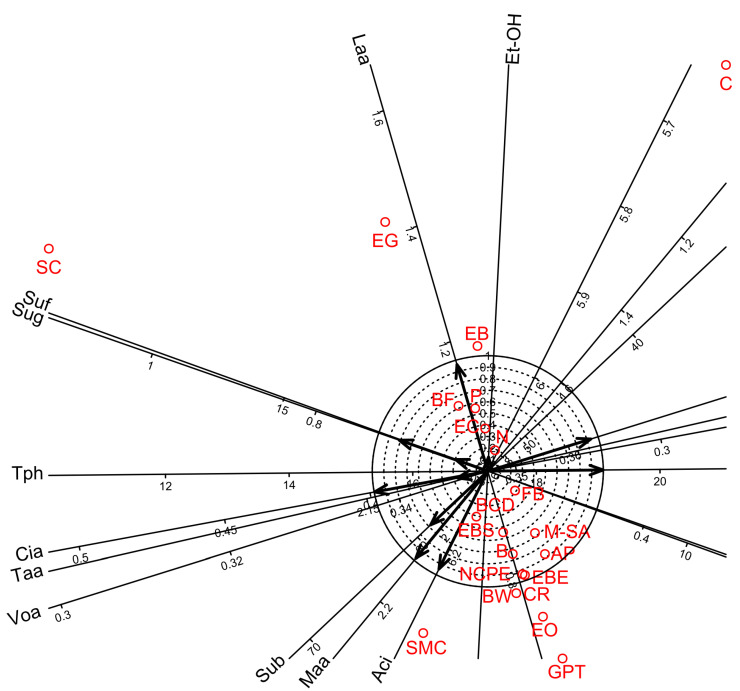
Biplot diagrams of Factor 1 versus Factor 2 of Principal Axis Factor Analysis (Iterated Principal Factor) conducted for ten oenological measurements of white wine samples in twenty-one bentonite product treatments (Debrecen-Pallag, Hungary). Oenological parameters are Et-OH: Ethyl-alcohol, Sug: Sugar, Aci: Acidity, Taa: Tartaric acid, Maa: Malic acid, Laa: Lactic acid, Cia: Citric acid, Voa: Volatile acid, Suf: Sulphur-free, Sub: Sulphur-bound, Tph: Total phenol. Explanations for bentonite product name abbreviations (red colour) are given in Table 1.

Factor analyses for the oenological parameters showed that four factors were sufficient to account for 92.0% of the total variance. Factor 1 accounted for 45.4% of the total variance (Table 9) and showed strong significant correlations for seven parameters. Among these seven parameters, five showed high positive loadings (sugar, acidity, malic acid, citric acid, and sulphur-bound) while two showed high negative loadings (volatile acid and total phenol). Factors 2, 3, and 4 accounted for 22.7, 14.4, and 9.5% of the total variance, respectively (Table 9) and showed significant positive correlations for lactic acid, alcohol, and sulphur-free, respectively. In addition, biplot diagrams of Factor 1 vs. Factor 2 visualised the above relationship among the oenological measurements and the bentonite product treatments (Figure 1).

#### 3.5.2. Pearson Correlation and Factor Analyses for Chemical Elements

When the data set of the 19 chemical elements of white wine were analyzed for all bentonite products together, 190 parameter pairs were correlated. Of the 190 pairs, 51 correlated significantly at *p* = 0.05 probability level (Table 10).

Among these 51 pairs, 50 were correlated positively (Al vs. Na, Al vs. Cr, Al vs. Fe, Al vs. Sr, Al vs. Ba, Al vs. Pb, B vs. Ca, B vs. K, B vs. Mg, B vs. P, B vs. Cr, B vs. F, B vs. Co, B vs. Ni, B vs. Cd, Ca vs. K, Ca vs. Mg, Ca vs. Cr, Ca vs. Cr, Ca vs. Fe, Ca vs. Co, Ca vs. Cd, K vs. Mg, K vs. Sr, K vs. Cd, Mg vs. Na, Mg vs. P, Mg vs. Cr, Mg vs. Fe, Mg vs. Co, Mg vs. Ni, Na vs. Cr, Na vs. Fe, Na vs. Co, Na vs. Ni, Na vs. Ba, Na vs. Pb, P vs. Zn, S vs. Mn, S vs. Sr, Cr vs. Fe, Cr vs. Co, Cr vs. Ni, Cr vs. Ba, Mn vs. Sr, Fe vs. Co, Fe vs. Ni, Fe vs. Ba, Co vs. Ni, Ni vs. Cd, Sr vs. Cd, and Ba vs. Pb) and one negatively (Al vs. Cu), indicating a strong connection among most chemical elements (Table 10).

**Figure 2:**
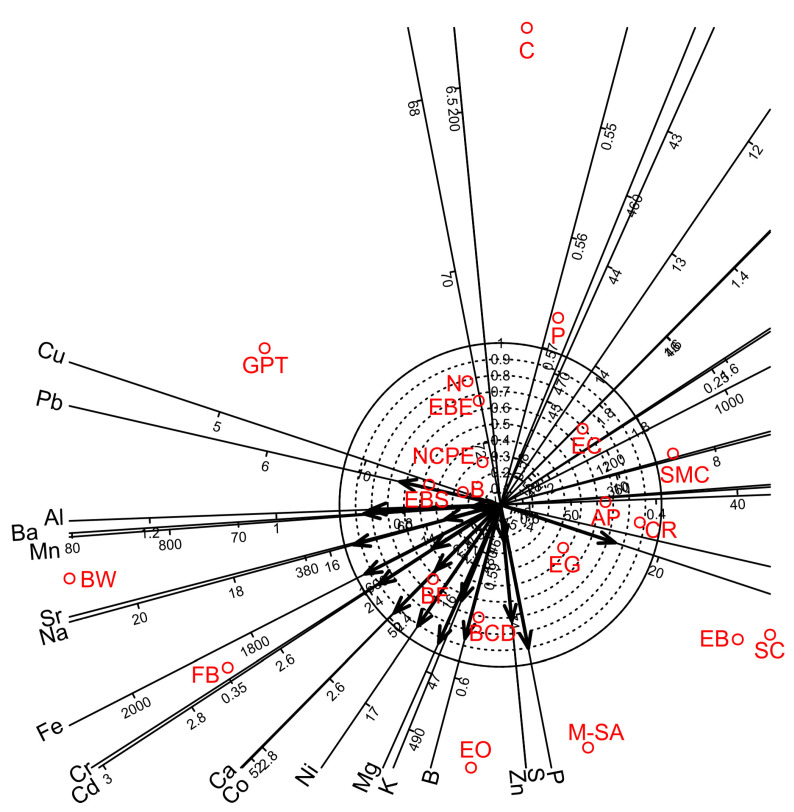
Biplot diagrams of Factor 1 versus Factor 2 of Principal Axis Factor Analysis (Iterated Principal Factor) conducted for nineteen chemical elements of white wine samples in twenty-one bentonite product treatments (Debrecen-Pallag, Hungary). Explanations for bentonite product name abbreviations (red colour) are given in Table 1.

Factor analyses for the nineteen chemical elements of white wine showed that four factors were sufficient to account for 81.5% of the total variance. Factors 1, 2, 3, and 4 accounted for 48.8, 16.3, 11.1, and 5.4% of the total variance, respectively (Table 11). Factors 1–3 provided separate groups of chemical elements. Factor 1 gave high positive loadings for B, Ca, K, Mg and P; Factor 2 showed high positive loadings for Mn, Sr and Cd; and Factor 3 gave high positive loadings for Co, Ni, Fe, Cr and Na. Factor 4 partially overlapped with Factor 3 and gave high positive loadings for Fe, Cr, Na, Ba, Pb, Al and Cu. Biplot diagram prepared for Factor 1 *vs* Factor 2 visualised strong relationships among the chemical elements and the bentonite products (Figure 2).

#### 3.5.3. Pearson Correlation and Factor Analyses for Volatile Organic Compounds (VOCs)

When the data set of the twenty-one VOCs of the white wine samples was analyzed for all bentonit products together, 214 parameter pairs were correlated. Of the 214 pairs, 134 correlated significantly at *p* = 0.05 probability level (Table 12). All the 134 pairs were correlated positively, indicating a strong connection among most VOCs (Table 12).

**Figure 3:**
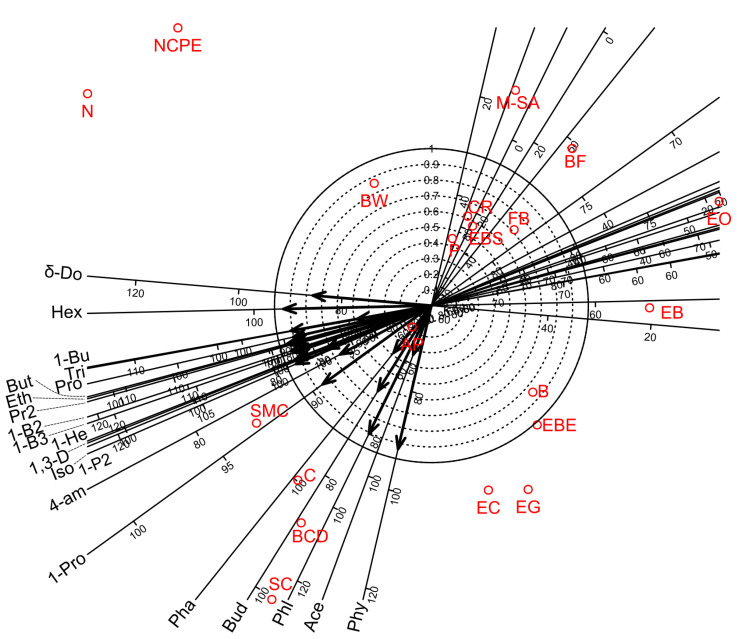
Biplot diagrams of Factor 1 versus Factor 2 of Principal Axis Factor Analysis (Iterated Principal Factor) conducted for twenty-two volatile organic compounds (VOCs) of white wine samples in twenty-one bentonite product treatments (Debrecen-Pallag, Hungary). Explanations for VOCs abbreviations are given in Table 12. Explanations for bentonite product name abbreviations (red colour) are given in Table 1.

Factor analyses for the twenty-one VOCs of wine showed that four factors were sufficient to account for 86.9% of the total variance. Factors 1, 2, 3, and 4 accounted for 67.3, 9.6, 5.6, and 4.5% of the total variance, respectively (Table 13). Factor 3 provided a separate group of VOCs while Factors 1 and 4 partially overlapped with Factor 2. Factor 1 gave high positive loadings for 13 VOCs (1-Propanol, Ethyl Acetate, 1-Propanol, 2-methyl-, Propanoic acid, ethyl ester, 1,3-Dioxolane, 2,4,5-trimethyl-, 1-Butanol, 3-methyl-, 1-Butanol, 2-methyl-, (S)-, Propanoic acid, 2-methyl-, ethyl ester, Butanoic acid, ethyl ester, 1-Hexanol, 1-Butanol, 3-methyl-, acetate, Hexanoic acid, ethyl ester and Octanoic acid, ethyl ester). Factor 2 showed high positive loadings for 7 VOCs (1-Butanol, 3-methyl-, Butanoic acid, ethyl ester, 1-Hexanol, 2-Butanone, Triethyl borate, Phenylethyl Alcohol, and Butanedioic acid, diethyl ester). Factors 3 and 4 gave high positive loadings for two (Phenol, 2-methyl-, Phenol, 2-methoxy-) and two (Phenylethyl Alcohol, Acetic acid) VOCs, respectively. The biplot diagram for the first two factors also demonstrated the above relationships among the VOCs measurements and the bentonite product treatments (Figure 3).

#### 3.5.4. Pearson Correlation and Factor Analyses for Organoleptic Parameters

When the data set of the 10 organoleptic parameters of wine were analyzed for all bentonit products together, 46 parameter pairs were correlated. Of the 46 pairs, 14 correlated significantly at *p* = 0.05 probability level (Table 14).

All these 14 pairs were correlated positively (clearness vs. colour, flavour intensity vs. flavour character, flavour intensity vs. flavour quality, flavour character vs. flavour quality, flavour character vs. overall harmony, flavour quality vs. taste intensity, flavour quality vs. overall harmony, taste intensity vs. taste character, taste intensity vs. taste quality, taste intensity vs. taste persistency, taste intensity vs. overall harmony, taste character vs. taste persistency, taste quality vs. overall harmony, and taste persistency vs. overall harmony) (Table 14).

**Figure 4:**
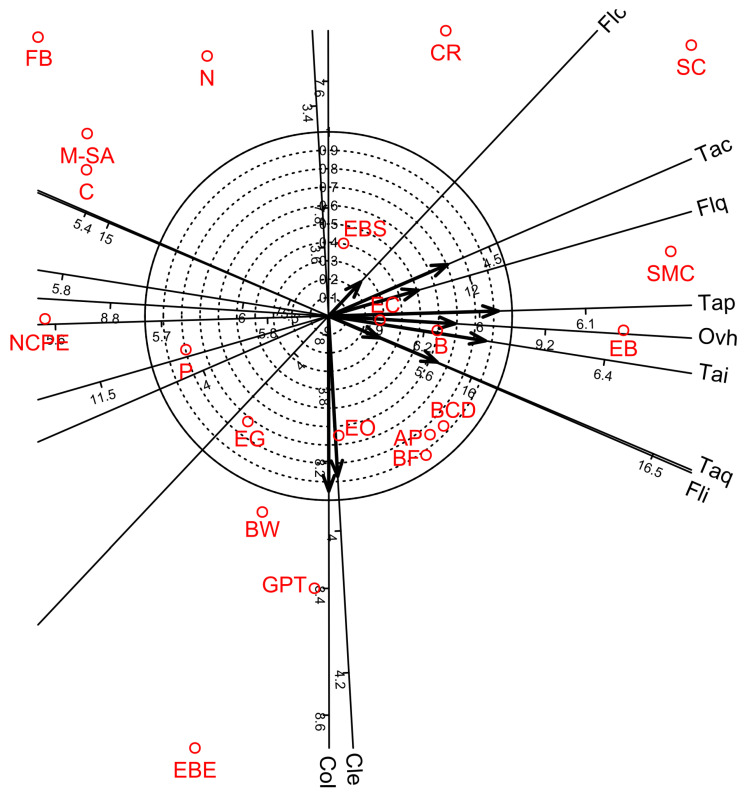
Biplot diagrams of Factor 1 versus Factor 2 of Principal Axis Factor Analysis (Iterated Principal Factor) conducted for ten organoleptic parameters of white wine samples in twenty-one bentonite product treatments (Debrecen-Pallag, Hungary). Organoleptic parameters are Cle: Clearness, Col: Colour, Fli: Flavor intensity, Flc: Flavor character, Flq: Flavor quality, Tai: Taste intensity, Tac: Taste character, Taq: Taste quality, Tap: Taste persistency, Ovh: Overall harmony. Explanations for bentonite product name abbreviations (red colour) are given in Table 1.

Factor analyses for the 10 organoleptic parameters of wine showed that four factors were sufficient to account for 87.5% of the total variance. Factors 1, 2, 3, and 4 accounted for 47.2, 21.2, 11.3, and 8.0% of the total variance, respectively (Table 15). Factors 1–3 provided separate groups of organoleptic parameters. Factor 1 gave high negative loadings for flavour quality, taste intensity, taste character, taste quality, taste persistency and overall harmony; Factor 2 showed high positive loadings for clearness and colour; and Factor 3 gave high positive loadings for flavour intensity and flavour character. Factor 4 overlapped with Factor 1 and gave high negative loadings for taste quality. In addition, biplot diagram of Factor 1 vs. Factor 2 visualised the distribution among the organoleptic parameters and the bentonite product treatments (Figure 4).

## 4. Discussion

Our study demonstrated that several oenological parameters, elemental compositions, aroma compounds and organoleptic parameters of white wine were affected by the selected eighteen bentonite products as fining agents. In addition, Pearson correlation and factor analyses demonstrated large numbers of significant intercorrelations among oenological, elemental, volatile, and organoleptic properties.

Our study confirmed that most of the bentonite products affected most of the oenological parameters of white wine (Table 3). In addition, this study showed that none of the bentonite products differed from the control treatment for the contents of ethyl-alcohol, tartaric acid and volatile acid in the wine samples (Table 3). Our results were confirmed by previous studies [16,37] in the case of ethyl-alcohol and volatile acidity contents, and the obtained values of ethyl-alcohol and volatile acidity contents were in the ranges given by the wine production regulations [44]. In addition, similar ranges of ethyl-alcohol, total acidity and volatile acidity values were reported by other white wine studies [45,46], where no bentonite fining was applied. Cheng and Watrelot [15] also reported that titratable acidity, ethanol, and tartaric acid contents were not different between the bentonite and control treatments during wine bottling. However, in contrast with our results, the study of Maslov-Bandic et al. [16] demonstrated that the sugar content of wine samples was significantly lower in the bentonite treatments compared to the control. In addition, different results were also reported by Cheng and Watrelot [15], who showed that malic acid contents were significantly lower in the bentonite treatment compared to the control treatment during wine bottling. The different results of previous studies from this study may be due to the fact that various bentonite products, various geographical origins of grape productions, various grape cultivars and/or various analytical methods were used in previous bentonite fining studies. Although various bentonite products can cause differences in oenological parameters, this bentonite fining study showed that the relationships are strong among most of the oenological parameters independently on bentonite products (18 pairs out of 55 ones were significant in correlation analyses and seven parameters out of 10 showed strong significant correlations for Factor 1, Table 8 and Table 9). This clearly indicates that various bentonite products not only change single oenological parameters but that strong intercorrelative changes can be expected among the parameters (e.g., among sugar, acidity malic acid, citric acid, volatile acid and total phenol) if we apply bentonite finings.

Similar to several previous studies, results of this work showed that bentonite fining affects the amounts of macro- and micro-elements (Table 4 and Table 5). The bentonite products, applied in the wine samples, caused changes in the amounts of Al, Ba, Ca, Cu, Fe, K, Mg, Mn, Na, Ni and Pb in this study (Table 4 and Table 5), which were in agreement with several previous studies [23,27,46,47,48,49,50,51,52,53]. Bentonite fining of wine increased the amounts of Na, Al and Ca in previous studies (e.g., [10,17,18,19,20]), which corresponded well to results of this study. In addition, Fe, Sr and Ba contents were also shown to increase in red wine samples by bentonite fining [21], which was also confirmed by this study in white wine samples. Previous wine studies showed that Cu, K and Zn contents [22] and also B content [23] decreased in the bentonite treatments compared to the control. The decrease of K and Zn contents by bentonite fining was not confirmed by this study, as most bentonite products caused a significant increase of K and Zn contents in the white wine samples (Table 4 and Table 5). In addition, the decrease of B content by bentonite fining was not in line with this study, as similar B quantities were measured in both the bentonite-treated and nontreated white wine samples (Table 4). The largest decrease was achieved for Cu (−43%) in the study of Nicolini et al. [22], which was confirmed by this study for the bentonite products of NCPE and GPT (Table 5). The different results of previous studies from this study may be due to the fact that various concentrations of bentonite products are applied and/or the used bentonite products contained various amounts of mineral composition, which contaminated differently the treated white samples. In addition, this bentonite fining study showed that the relationships among elements are clustered in Factors 1, 2, 3, and 4 (e.g., in Factor 1, B, Ca, K, Mg and P or in Factor 3, Co, Ni, Fe, Cr and Na, Table 11). This suggests that various bentonite products can cause intercorrelative changes among attached elemental groups in various level if bentonite fining is applied.

This study showed that bentonite fining significantly decreased the amounts of several VOCs in white wine samples compared to the control treatment but that the level of decrease was dependent on the types of VOCs and bentonite products (Table 6). On the other hand, bentonite fining was able to increase significantly several VOCs depending on the types of bentonite products (Table 6). The recent study of Horvat et al. [14] also demonstrated that bentonite negatively affected wine quality by changing some key fermentation volatiles compared to treatments without bentonite fining. Some previous studies showed that bentonite fining may reduce aroma and flavour compounds in the wine due to direct adsorption and deproteinization [28,29,30,31,32,33]. The aroma loss was verified after multiple treatments in various wine types [32,34]. Lira et al. [37] showed that the possible loss of volatile compounds may be due to adsorption on bentonite. The results of Sanborn et al. [35] also indicated that fining agents can perform unpredictably and may result in various levels of wine-quality reductions [35]. This phenomenon was supposed to be due to the fact that bentonite can connect with volatile components by chemical bindings such as van der Waals or hydrogen bonds [32,54,55,56]. In addition, this study showed that intercorrelations among many VOCs are strong already in the Factor 1, which accounted for a large amounts of variance (Table 13, Figure 3). This phenomenon indicates that bentonite fining has a strong influence on most of the VOCs independently of the type of the bentonite products used.

Several studies showed that the loss of aroma compounds in wine after bentonite treatments severely affects the sensory attributes of the wine too [28,29,32,33,34]. In this study, the organoleptic parameters were variously affected by bentonite fining compared to the control treatment; for example, organoleptic parameters were not affected (e.g., EBS vs. all organoleptic parameters), or were significantly decreased (e.g., EBE vs. flavour intensity) or significantly increased (e.g., EBE vs. colour) by various bentonite products (Table 7). The various effects could be explained by the various origins of bentonite products which differently affected the taste parameters due to their various mineral compositions. In addition, this study also showed that some flavour and taste parameters are well attached to each other, for example in Factor 1 (Table 15). This phenomenon was true in the overall inter-correlation analyses of all bentonite products, which indicates that organoleptic parameters are highly affected by bentonite finings. These influences are strongly connected to the changes of quality compositions of the wine, which was due to the used bentonite product.

## 5. Conclusions

This study clearly showed that oenological parameters, elemental compositions, aroma compounds and organoleptic parameters of the white wine samples were affected variously by the selected eighteen bentonite products as fining agents. In addition, analyses of inter-correlations showed a large number of significant intercorrelations among individual properties of oenological, elemental, volatile, and organoleptic attributes.

Bentonite fining will affect several wine-quality attributes (such oenological, elemental, volatile and organoleptic) alone and also in association with each other, which should be considered when we select a bentonite product for fining. This suggests that the right choice of bentonite products is essential (e.g., in relation to type of wine, inner content of wine, geographical origin) but that complex and intercorrelative effects of bentonite fining on all quality attributes of the wine can be expected, whatever products are used for bentonite fining.

It is complicated to select one product which is generally suitable for bentonite fining. In our specific case, the AP treatment seemed to be the most suitable for bentonite fining of our white wine samples. However, this study also demonstrated that several bentonite products are suitable for good fine toning of the wine according to the quality attributes of the wine or in the practice of organoleptic evaluations. It needs to be emphasized that all these phenomenon cannot be simply deduced from the direct changes of elemental and/or direct or indirect change of volatile compositions and that the complexity of the changes always has to be taken into consideration.

## Figures and Tables

**Table 1:** Abbreviation, active ingredients, producer suggested dosage, applied dosage and dissolved oxygen at bottling of the bentonite products in order to evaluate white wine from Debrecen-Pallag, Hungary. Manufacturers, town and country of the applied products are Perdomini-IOC S.p.a., San Martino, Italy for E-Benton Super, Mixgel-SA, Caseo-star, ALFA-P, Bentamin-100, Pentagel, Bentonite-Compact Due and E-Benton Extra; Ever s.r.l., Venezia, Italy for Fort Benton, Everclar Compact, Everclar Beta, Nucleobent, Everclar Omega, Everclar Gamma and Benton flash; Erbslöh Geisenheim Gmbh, Geisenheim, Germany for Na-Calit Poretec Erbslöh and Granubent Pore-Tec; Süd-Chemie Verwaltungs GmbH, München, Germany for BW 200.

Product Name	Abbrev.	Active Ingredients	Producer Suggested Dosage (g/L)	Applied Dosage (g/L)	Dissolved Oxygen at Bottling (ppm)
Control	C	-	-	-	2.2
Sulfur Control	SC	10 mg/L sulfitization	-	-	2.4
Sulfur + Mix Control	SMC	10 mg/L sulfitization + intensive mixing	-	-	2.4
E-Benton Super	EBS	activated Na-bentonite	30–80	55	3.7
Fort Benton	FB	Na-bentonite	50–100	75	2.4
Mixgel-SA	M-SA	gelatine, kazeine, albumin, bentonite, colloidal silicium dioxide	40–50	45	1.9
Na-Calit Poretec Erbslöh	NCPE	Na- Ca-bentonite	50–150	100	2.4
Everclar Compact	EC	Na- Ca-bentonite	20–80	50	2.7
Caseo-star	CR	K-casein, activated bentonite	30–50	40	1.7
BW 200	BW	bentonite	40–120	80	2.4
Everclar Beta	EB	yeast cell wall, PVPP, silicate	5–10–20–30	17.5	1.8
ALFA-P	AP	gelatine, caseine, albumine, bentonite, colloidal silicium dioxide (“4”) + PVPP	30–80	55	2.6
Bentamin-100	B	bentonite, silicium dioxide	50–100	75	2.6
Nucleobent	N	bentonite	20–40	30	1.8
Granubent Pore-Tec	GPT	bentonite	-	-	2.1
Everclar Omega	EO	bentonite, fish glue,	20–50	35	2.2
Pentagel	P	activated Na-bentonite	10–20	15	1.9
Bentonite-Compact Due	BCD	Ca-, Na-bentonite	70–150	70	2.5
Everclar Gamma	EG	montmor., PVPP, selected silicate, special active carbon	30–50	40	2.0
E-Benton Extra	EBE	activated Na-bentonite	50–100	75	2.2
Benton flash	BF	activated Na-bentonite	20–80	50	2.5

**Table 2:** Molecular formula, chemical group and retention time (RT) for twenty-two volatile organic compounds (VOCs) determined by gas chromatography from the white wine samples in twenty-one bentonite product treatments including three control treatments (Debrecen-Pallag, Hungary).

VOCs	Molecular Formula	Chemical Group	RT
**Alcohols**			
1-Propanol	C_3_H_8_O	primary alcohols	3.318
1-Propanol, 2-methyl-	C_4_H_10_O	primary alcohols	4.396
1-Butanol, 3-methyl-	C_5_H_12_O	primary alcohols	6.464
1-Butanol, 2-methyl-, (S)-	C_5_H_12_O	primary alcohols	6.547
1-Hexanol	C_6_H_14_O	alcohol	9.160
**Esters**			
Ethyl Acetate	C_4_H_8_O_2_	carboxylic acid esters	4.182
Propanoic acid, ethyl ester	C_5_H_10_O_2_	carboxylic acid esters	6.012
Isobutyl acetate	C_6_H_12_O_2_	carboxylic acid esters	7.269
1-Butanol, 3-methyl-, acetate	C_7_H_14_O_2_	carboxylic acid esters	9.320
Propanoic acid, 2-methyl-, ethyl ester	C_6_H_12_O_2_	ethyl ester	6.982
Butanoic acid, ethyl ester	C_6_H_12_O_2_	ethyl ester	7.815
Hexanoic acid, ethyl ester	C_8_H_16_O_2_	ethyl ester	11.587
Butanedioic acid, diethyl ester	C_8_H_14_O_4_	fatty acid esters	14.582
Octanoic acid, ethyl ester	C_10_H_20_O_2_	fatty acid esters	14.867
Triethyl borate	C_6_H_15_BO_3_	ester	7.632
**Acids**			
4-Amino-1,5-pentandioic acid	C_5_H_9_NO_4_	alpha-amino acid	6.910
Acetic acid	C_2_H_4_O_2_	carboxylic acids	3.566
**Others**			
δ-Dodecalactone	C_10_H_18_O_2_	gamma butyrolactones	5.900
1,3-Dioxolane, 2,4,5-trimethyl-	C_6_H_12_O_2_	1,3-dioxolanes	6.380
Phenol, 2-methyl-	C_7_H_8_O	ortho cresols	12.635
Phenol, 2-methoxy-	C_7_H_8_O_2_	methoxyphenols	13.410
Phenylethyl alcohol	C_8_H_10_O	benzene	13.829

**Table 3:** Quantities of nine basic wine parameters (ethyl-alcohol, sugar, acidity, tartaric acid, malic acid, lactic acid, citric acid, volatile acid, and total phenol) in white wine samples treated with twenty-one bentonite products including control treatments (Debrecen-Pallag, Hungary). Different letters within each column are significantly different among the bentonite product treatments at *p* = 0.05 according to LSD t-tests (LSD_0.05_). ns = nonsignificant. Explanations for bentonite product name abbreviations are given in Table 1.

Product Name	Ethyl-Alcohol	Sugar	Acidity	Tartaric Acid	Malic Acid	Lactic Acid	Citric Acid	Volatile Acid	Total Phenol	Sulphur Free	Sulphur Bound
Abbreviation	*v/v*%	g/L	g/L	g/L	g/L	g/L	g/L	g/L	mg/L	mg/L	mg/L
C	12.11	0.5ab	5.60a	2.1	0.8a	1.6c	0.26a	0.39	21.0d	12cde	21a
SC	12.10	1.2e	6.13ab	2.2	2.1c	1.5c	0.49c	0.31	10.2a	21h	61fgh
SMC	12.13	0.6bc	6.20b	2.2	2.0bc	1.1b	0.41bc	0.34	15.6b	9bcd	77j
EBS	11.92	0.8d	6.01ab	2.0	2.0bc	0.8a	0.36ab	0.36	17.2bc	2a	58efg
FB	12.04	0.5ab	6.05ab	2.1	1.8b	0.9ab	0.35ab	0.35	17.7c	12ce	54def
M-SA	12.09	0.6bc	6.13ab	2.2	1.9bc	0.9ab	0.35ab	0.36	17.9c	14e	56def
NCPE	12.09	0.4a	6.14ab	2.1	1.9bc	0.9ab	0.36ab	0.35	17.5bc	13def	66ghi
EC	12.05	0.6bc	6.16b	2.1	1.8b	0.9ab	0.35ab	0.35	17.4bc	13def	53cdef
CR	12.1	0.6bc	6.16b	2.2	1.9bc	1.0ab	0.37ab	0.34	17.3bc	11cde	54def
BW	12.01	0.5ab	6.07a	2.1	1.8b	0.9ab	0.36ab	0.34	17.6c	20gh	69hij
EB	12.11	0.6bc	6.17b	2.2	1.9bc	0.9ab	0.34ab	0.35	17.5bc	6ab	35b
AP	12.09	0.5ab	6.15b	2.1	1.9bc	0.9ab	0.35ab	0.35	18.0c	17fgh	49cde
B	12.05	0.6bc	6.13a	2.1	1.8b	0.9ab	0.36ab	0.35	17.4bc	14ef	56def
N	12.07	0.5ab	6.17b	2.1	1.9bc	0.9ab	0.35ab	0.35	17.5bc	17fgh	52cdef
GPT	12.07	0.5ab	6.14ab	2.1	1.9bc	0.9ab	0.36ab	0.36	18.0c	8bcd	58efg
EO	12.08	0.6bc	6.13ab	2.2	1.9bc	0.9ab	0.36ab	0.35	17.8c	12cde	56def
P	12.10	0.6bc	6.17b	2.2	1.9bc	0.9ab	0.35ab	0.35	17.3bc	9bcd	43bc
BCD	12.06	0.5ab	6.14ab	2.2	1.8b	0.9ab	0.35ab	0.36	17.7c	14def	52cdef
EG	12.02	0.7cd	6.19b	2.1	1.9bc	0.9ab	0.35ab	0.35	16.4bc	8bc	47cd
EBE	12.07	0.5ab	6.14ab	2.1	1.9bc	0.9ab	0.36ab	0.35	17.6c	16fg	54def
BF	12.05	0.5ab	6.11ab	2.1	1.9bc	1.0ab	0.36ab	0.35	16.9bc	14ef	52cdef
LSD_0.05_	ns	0.18	0.54	ns	0.26	0.22	0.11	ns	1.9	4.1	9.5

**Table 4:** Quantities (mg/L) of eight macro- and meso elements (P, K, Ca, Mg, S, Al, Na, and B) in white wine samples treated with twenty-one bentonite products including control treatments (Debrecen-Pallag, Hungary). Different letters within each column are significantly different among the bentonite product treatments at *p* = 0.05 according to LSD t-tests (LSD_0.05_). Explanations for bentonite product name abbreviations are given in Table 1.

Product Name	P	K	Ca	Mg	S	Al	Na	B
C	66.7a	432.7a	41.22a	41.8a	5.845a	0.270a	7.75a	0.526a
SC	74.9cde	479.3b–e	41.87ab	45.7b	6.955def	0.296ab	8.82ab	0.586bc
SMC	73.6b–e	470.5bcd	45.81abc	45.0ab	6.563a–e	0.302ab	8.63ab	0.572bc
EBS	71.5bc	488.0cde	50.75cde	46.3b	7.358fg	0.918gh	14.28cde	0.585bc
FB	74.1cde	474.2b–e	48.12cd	47.3b	6.338a–d	0.720ef	17.97e	0.587bc
M-SA	74.9cde	486.5b–e	50.85cde	46.8b	7.811gh	0.465b	11.05a–d	0.603c
NCPE	72.8b–e	485.5b–e	50.02cde	46.0b	9.181i	1.130i	12.97bcd	0.596bc
EC	72.5b–e	474.3b–e	48.45cde	45.7b	6.525a–e	0.701ef	10.09abc	0.578bc
CR	71.9bcd	491.5de	49.80cd	45.0ab	7.198ef	0.375abc	10.31a–d	0.588bc
BW	72.6b–e	488.8cde	51.81de	46.8b	7.363fg	1.465j	21.21f	0.599c
EB	75.4de	480.1b–e	48.05cd	45.9b	6.213abc	0.299ab	8.945ab	0.591bc
AP	72.7bcd	479.7b–e	50.67cde	45.7b	8.438hi	0.554de	11.12a–d	0.592bc
B	72.3b–e	475.8b–e	49.77cde	45.8b	6.281a–d	0.555de	11.73a–d	0.581bc
N	73.4b–e	466.3bc	47.95cd	45.5b	6.811b–f	0.739f	12.71bcd	0.574bc
GPT	70.2ab	496.2e	47.02bcd	45.8b	6.538a–e	1.088hi	17.92ef	0.588bc
EO	75.9e	477.7b–e	47.55cd	47.2b	6.703b–f	0.550cde	13.67b–e	0.595bc
P	70.1ab	463.5bcd	47.65cd	44.4b	6.091ab	0.419a–d	10.15abc	0.565b
BCD	72.9b–e	487.2cde	53.30e	46.7b	7.865gh	0.762fg	12.25a–d	0.595bc
EG	72.8b–e	478.5b–e	48.85cde	45.8b	6.138abc	0.427a–d	11.21a–d	0.584bc
EBE	71.8bcd	474.3b–e	47.70cd	45.4b	8.283hi	0.764fg	13.51b–e	0.575bc
BF	74.1cde	474.5b–e	49.07cde	46.5b	6.823c–f	0.752fg	15.15de	0.585bc
LSD_0.05_	3.74	23.41	5.21	3.38	0.731	0.175	4.93	0.075

**Table 5:** Quantities (µg/L) of eleven micro elements (Cr, Mn, Fe, Co, Ni, Cu, Zn, Sr, Cd, Ba, and Pb) in white wine samples treated with twenty-one bentonite products including control treatments (Debrecen-Pallag, Hungary). Different letters within each column are significantly different among the bentonite product treatments at *p* = 0.05 according to LSD t-tests (LSD_0.05_). Explanations for bentonite product name abbreviations are given in Table 1. Order of used letter for significance differences: a, b, c, d, e, f, g, h, i, j, k, l, m.

Product Name	Cr	Mn	Fe	Co	Ni	Cu	Zn	Sr	Cd	Ba	Pb
C	1.58a	725.7ab	1047a	1.56a	12.5a	14.2fgh	209.5a	316.2a	0.198a	39.67a	2.27a
SC	1.71abc	747.1abc	1111abc	1.69ab	14.7a–d	23.2m	248.2cde	337.5ab	0.243ab	44.18a–d	2.85abc
SMC	1.63ab	741.2abc	1105ab	1.72abc	13.7ab	16.4hij	246.0cde	340.5abc	0.225ab	48.05c	2.63ab
EBS	2.36f–i	778.7bcd	1416g	2.02de	14.7a–d	11.3cde	245.7cde	376.3c–g	0.301abc	67.35h	4.24hi
FB	2.85j	792.9cde	1728k	2.86h	17.9e	10.9bcd	253.2cde	363.5b–g	0.291abc	63.02gh	4.07gh
M-SA	2.24fgh	744.2abc	1474ij	2.41fg	16.2b–e	12.9defg	263.5e	350.5a–f	0.323bcd	42.93ab	2.81ab
NCPE	2.38hi	852.5f	1469ij	2.05de	15.4a–e	8.81b	252.7cde	431.3i	0.311bcd	55.85ef	4.72ij
EC	1.86a–e	826.1de	1256b–f	1.80abcd	14.2ab	11.6cd	236.0a–d	386.3fgh	0.278ab	54.11e	3.46def
CR	1.93b–e	764.2abc	1220b–e	2.02de	15.7b–e	18.6jkl	244.7b–e	371.0b–g	0.413d	47.78bcd	3.49d–g
BW	2.59ij	781.2cd	2132l	2.82h	16.1b–e	9.41bc	228.8abc	357.5b–g	0.318bcd	74.32i	6.13k
EB	1.76a–d	769.5abc	1116abc	1.92bcd	14.3a–d	18.9k	258.2de	349.3a–e	0.225ab	45.10bcd	2.51ab
AP	2.12efg	886.2f	1274d–g	1.94bcd	14.9a–d	19.3l	248.3cde	388.2fgh	0.290abc	45.62bcd	3.79e–h
B	2.33f–i	769.0abc	1440hij	2.24ef	15.8b–e	13.6efg	250.0cde	379.8d–g	0.315bcd	53.88e	4.78ij
N	1.83a–e	759.5abc	1259c–f	1.84acd	14.4a–d	11.4cde	244.7b–e	359.3b–g	0.251ab	66.67h	9.46m
GPT	2.42hi	849.0f	1586jk	1.99cde	16.0b–e	6.42a	236.5a–e	421.7hi	0.385cd	81.95j	7.68l
EO	2.39ghi	788.9bcd	1598jk	2.57gh	17.3de	16.9ijk	356.5de	359.8b–g	0.278ab	56.25ef	3.36cde
P	1.75a–d	765.2abc	1132a–d	1.73abc	14.1ab	15.2ghi	232.7a–d	341.2abc	0.235ab	43.25abc	2.98bcd
BCD	2.22fgh	774.1bcd	1606jk	2.46fg	15.8b–e	11.5cde	257.5de	394.5ghi	0.391cd	56.35ef	6.20k
EG	2.07d–g	718.5a	1315e–h	1.98cde	14.5a–d	13.2def	217.5ab	336.3ab	0.245ab	43.25abc	2.51ab
EBE	2.02c–f	846.5ef	1291e–h	2.06de	14.3abc	12.1def	251.2cde	382.0eg	0.313bcd	48.70d	5.16j
BF	2.14e–h	774.0bcd	1380f–i	2.26e	17.2cde	12.3def	250.3cde	373.8b–g	0.321bcd	60.71fg	3.96fgh
LSD_0.05_	0.34	54.9	151	0.28	2.93	2.32	27.3	38.1	0.105	4.92	0.58

**Table 6:** Percentage contribution of twenty-two volatile organic compounds (VOCs) to control (C) treatment in white wine samples treated with twenty-one bentonite products including control treatments (Debrecen-Pallag, Hungary). The order of VOCs follows the increasing retention time (see Table 2), which corresponds to the elongation of the carbonic chain. The relative presence of VOCs is expressed in the percentage of control (C) treatment. Different letters within each column are significantly different among the bentonite product treatments at *p*= 0.05 according to LSD t-tests (LSD_0.05_). Order of used letter for significance differences: a, b, c, d, e, f, g, h, i, j, k, l, m. ‘-‘: the VOCs is not detected in the treatment. Explanations for bentonite product name abbreviations are given in Table 1.

**Product Name**	**1-Propanol**	**1-Propanol, 2-Methyl-**	**1-Butanol, 3-Methyl-**	**1-Butanol, 2-Methyl-, (S)-**	**1-Hexanol**	**Ethyl** **Acetate**	**Propanoic Acid, Ethyl Ester**	**Isobutyl Acetate**	**1-Butanol, 3-Methyl-, Acetate**	**Propanoic Acid, 2-Methyl-, Ethyl Ester**	**Butanoic Acid, Ethyl Ester**
C	100e	100cd	100efg	100e	100hi	100e–h	100fgh	100d–h	100d–g	100hi	100de
SC	94.9cde	104.3e	114.6g	111.6g	109.5i	101.2fgh	89.7e–h	107.7fgh	92.9c–g	84.6f–i	101.0de
SMC	92.3cde	103.6de	105.2fg	104.9fg	73.4d–h	102.7gh	99.9fgh	112.7gh	108.3fg	86.5ghi	108.3e
EBS	79.7b–e	90.9b–e	82.1def	80.2b–e	53.3a–f	90.4c–h	78.5c–f	87.2c–h	89.2c–f	71.2b–i	90.6cde
FB	76.1bcd	82.6ab	61.3a–d	65.7abc	31.9ab	79.2abc	57.5abc	67.3abc	76.4bcd	50.8a–e	68.6abc
M-SA	81.4b–e	79.4ab	69.5a–d	71.6abc	54.5a–f	83.1b–e	70.5b–e	92.6c–h	80.5b–e	68.5b–i	69.9abc
NCPE	89.6cde	101.3d	103.9fg	103.1fg	85.5f–i	99.3d–h	104.0h	114.1h	89.8c–f	79.0c–i	96.4de
EC	84.1b–e	97.5cde	77.5b–e	79.7b–e	63.7b–g	89.6c–h	73.6b–e	80.6b–f	79.2b–e	55.4a–g	82.1bcd
CR	85.0b–e	83.0ab	77.1b–e	77.4a–d	51.4a–f	88.2c–g	67.8bcd	99.0d–h	77.4bcd	50.3a–e	81.2bcd
BW	79.9b–e	92.6b–e	92.9ef	92.7def	77.2d–i	93.5c–h	83.5d–h	54.3ab	79.3b–e	81.4d–i	91.8de
EB	84.1b–e	73.6a	52.2a	56.9a	24.2a	69.6ab	57.7abc	82.9c–g	62.8ab	40.7ab	57.8ab
AP	83.2b–e	92.4b–e	94.8efg	93.7d–g	82.5e–i	94.8c–h	80.5def	91.2c–h	83.1b–f	60.5a–g	88.1cde
B	75.9bcd	80.6ab	64.3a–d	66.5abc	50.2a	82.5bcd	55.4ab	75.4b–e	71.2ab	48.2a–d	77.8bcd
N	80.0b–e	99.6cde	96.2efg	99.7efg	84.4f–i	102.8gh	88.5e–h	105.7e–h	117.3g	83.5e–i	110.4e
GPT	49.3a	–	94.8efg	86.1c–g	99.0hi	106.5h	102.1gh	75.2bcd	104.2efg	101.8i	109.1e
EO	65.4ab	72.2a	54.7ab	58.9a	26.1a	63.2ab	44.1ab	37.5a	47.6a	28.2a	47.4a
P	74.4bc	80.4ab	67.6a–d	70.6abc	46.7a–d	83.9b–f	71.3b–e	79.9b–f	75.8bcd	52.1a–f	77.8bcd
BCD	97.2de	102.2de	109.1g	108.4g	97.6ghi	100.9fgh	80.4df	112.3gh	96.7c–g	81.6d–i	95.9de
EG	82.9b–e	86.2abc	64.3a–d	68.8abc	43.4a–d	82.6b–e	75.3b–e	96.4c–h	71.7abc	46.8abc	76.8bcd
EBE	82.5b–e	89.6bcd	80.5cde	81.5b–e	67.1c–h	86.8b–g	80.9d–g	96.7c–h	75.7bcd	66.9b–h	85.8cde
BF	77.4bcd	79.4ab	57.6abc	62.5ab	34.8abc	78abc	63.7a–d	87.1c–h	70.9abc	44.4ab	65.7abc
LSD_0.05_	22.4	14.2	23.1	20.5	34.1	17.4	21.5	30.4	26.1	33.7	25.8
**Product Name**	**Hexanoic Acid, Ethyl Ester**	**Butanedi-oic Acid, Diethyl Ester**	**Octanoic Acid, Ethyl Ester**	**Triethyl Borate**	**4-Amino-1,5-Pentandioic Acid**	**Acetic Acid**	**δ-Dodecalactone**	**1,3-Dioxolane, 2,4,5-Trimethyl-**	**Phenol, 2-Methyl-**	**Phenol, 2-Methoxy-**	**Phenylethyl Alcohol**
C	100ghi	100f	100fgh	100fgh	100f	100e–h	100ef	100hi	100def	100fgh	100jk
SC	95.4e–h	80.8ef	94.2e–h	64.6b–e	81.4ef	82.3c–g	93.3def	90.3ghi	105.8ef	123.1h	102.8ijk
SMC	97.7fgh	93.9ef	106.1gh	68.1c–f	80.2def	118.6ghi	72.1b–e	86.2fgh	110.9ef	89.1e–h	90.2f–k
EBS	75.4b–g	89.6ef	78.4c–g	85.7efg	28.4a	91.9c–h	48.7ab	34.7ab	70.6bcd	54.8a–e	126.1k
FB	66.5a–d	–	38.2a	45.4abc	38.4ab	57.8a–d	–	41.2ab	–	37.3ab	27.1abc
M-SA	67.9a–e	53.8cd	70.6b–f	85.3efg	39.7ab	74.1b–f	41.7ab	46.9abc	–	23.4a	65.1d–h
NCPE	107.1hi	37.5abc	110.1h	104.3fgh	65.1b–e	55.7a–d	134.1g	76.5d–h	–	19.0a	25.9ab
EC	79.5c–h	73.8de	65.9a–d	129.6h	58.9a–d	97.7e–h	30.1a	59.3b–f	52.4abc	102.6fgh	60.5b–f
CR	74.1b–g	51.4bcd	72.4b–f	85.9efg	45.6abc	169.8j	115.9fg	58.6b–f	32.6a	49.7a–d	82.5e–i
BW	90.7d–h	79.5ef	88.3e–h	110.1fgh	65.4b–e	107.5fgh	60.5a–d	69.7c–g	–	56.9a–e	103.7ijk
EB	51.0ab	–	47.3ab	35.8ab	27.8a	65.4a–e	42.4ab	38.7ab	–	43.6abc	24.2a
AP	71.0a–f	74.4de	94.8e–h	109.4fgh	63.4b–e	150.6ij	110.1f	59.7b–f	50.9abc	79.9c–g	67.6e–i
B	74.9b–g	23.9a	57.8a–d	64.5b–e	47.7a–d	85.4c–h	48.1ab	46.6abc	50.1ab	74.6b–f	52.2a–e
N	126.4i	–	94.7efgh	88.9efg	75.7c–f	57.9a–d	113.1fg	77.6e–h	32.3a	28.4a	98.5g–k
GPT	81.1c–h	49.4bc	107.9gh	78.4def	99.9f	91.7c–h	97.8e	117.1i	86.2de	117.7gh	44.3a–d
EO	45.9a	–	–	37.9abc	75.6c–f	54.1abc	–	21.8a	–	18.5a	19.4a
P	70.2a–f	–	74.3b–f	50.2a–d	37.7ab	29.9ab	57.1abc	50.8b–e	39.9ab	39.9ab	86.1e–j
BCD	87.2d-h	77.5ef	87.4d–h	124.6h	70.7b–f	93.4d–h	85.5c–f	77.2e–h	123.1f	107.1fgh	62.3c–g
EG	71.4a–f	28.4ab	49.3abc	31.3a	43.9abc	72.7b–f	–	49.4a–d	50.1ab	88.4d–h	51.1a–e
EBE	75.1b–g	97.8f	82.8d–h	110.8gh	65.1b–e	121.1hi	55.1abc	53.9b–e	83.4cde	89.1e–h	120.9jk
BF	57.2abc	25.9a	65.2a–d	58.9a–e	28.4a	40.5ab	40.5ab	35.2ab	–	24.6a	29.2a–d
LSD_0.05_	27.8	23.1	29.8	32.2	33.1	38.2	33.2	27.7	32.7	38.8	36.2

**Table 7:** Effect of twenty-one bentonite product treatments including controls on ten organoleptic parameters in white wine samples (Debrecen-Pallag, Hungary). Different letters within each column are significantly different among the bentonite product treatments at *p* = 0.05 according to LSD t-tests (LSD_0.05_). Explanations for bentonite product name abbreviations are given in Table 1. Scales for the 10 parameters including the two anchors were as follows: skin and flesh colour: 0 = unacceptable, 9 = excellent; flavour intensity and character: 0 = very weak, 8 = very strong; flavour quality: 0 = unacceptable and 15 = excellent; taste intensity, character and persistency: 0 = very bad, 8 = excellent; taste quality: 0 = very bad, 20 = excellent and overall harmony: 0 = unacceptable, 10 = excellent.

Product Name	Clearness	Colour	Flavor Intensity	Flavor Character	Flavor Quality	Taste Intensity	Taste Character	Taste Quality	Taste Persistency	Overall Harmony
C	3.62bcd	7.69ab	5.46bcd	4.31de	12.00cde	6.08a–d	3.92ab	15.08abc	5.62a	8.85abc
SC	3.46abc	7.54a	5.85de	4.23cde	12.31de	6.46d	5.77f	15.38a–d	6.23c	9.08abc
SMC	3.77cde	7.85abc	5.15ab	3.85abc	11.85cde	6.31cd	4.46de	16.92f	6.15bc	9.31bc
EBS	3.69bcd	7.85abc	5.85de	4.00bcd	11.85cde	6.00a–d	4.00abc	14.85ab	5.92abc	8.85abc
FB	3.23a	7.54a	5.54bcd	4.08bcd	11.38abc	5.69ab	4.08a–d	14.62a	5.62a	8.77ab
M-SA	3.46abc	7.69ab	4.77a	3.77ab	10.77ab	5.62a	4.08a–d	15.00ab	5.62a	8.77ab
NCPE	3.54a–d	8.00a–d	5.54bcd	4.00bcd	11.69cde	5.69ab	4.00abc	15.54a–e	5.62a	8.85abc
EC	3.69bcd	8.00abc	5.08ab	3.85abc	11.54bcd	6.08a–d	3.92ab	14.85ab	5.92abc	8.77ab
CR	3.23a	7.54a	5.54bcd	4.08bcd	11.85cde	6.15bcd	4.31cde	15.08abc	6.00abc	9.00abc
BW	3.77cde	8.31bcd	5.92de	4.23cde	12.00cde	6.08a–d	4.23b–e	15.77b–e	5.85abc	9.08abc
EB	3.69bcd	8.00a–d	5.92de	4.08bcd	12.46e	6.38cd	4.46de	16.46ef	6.15bc	9.31bc
AP	3.77cde	8.15a–d	5.77cde	4.52e	12.46e	6.38cd	4.38cde	16.46ef	6.00bc	9.54c
B	3.62bcd	8.00a–d	5.31bc	3.85abc	11.83cde	6.38cd	4.46de	16.23def	5.85abc	9.15abc
N	3.38ab	7.54a	5.54bcd	3.85abc	12.00cde	5.92abc	3.92ab	15.54a–e	5.69abc	8.92abc
GPT	3.77cde	8.46cd	5.38bc	4.00bcd	12.15cde	6.15bcd	4.23b–e	15.77b–e	5.85abc	9.00abc
EO	3.85de	8.15a–d	5.92de	4.00bcd	11.85cde	6.15bcd	4.00abc	16.00c–f	5.85abc	9.15abc
P	3.85de	8.00a–d	5.46bcd	4.00bcd	12.31de	6.08a–d	4.08a–d	14.85ab	5.69abc	8.77ab
BCD	3.85de	8.15a–d	5.46bcd	3.77ab	11.69cde	6.23cd	3.92ab	15.54a–e	5.92abc	9.00abc
EG	3.69bcd	8.15a–d	5.46bcd	4.08bcd	11.54bcd	6.08a–d	4.08a–d	16.23def	5.77abc	9.15abc
EBE	4.62f	8.62d	5.31bc	3.54a	10.62a	5.92abc	3.77a	14.62a	5.69abc	8.46a
BF	4.08e	8.15a–d	6.15e	4.23cde	11.85cde	6.15bcd	4.54e	16.92f	6.00abc	9.23bc
LSD_0.05_	0.36	0.68	0.46	0.42	0.88	0.51	0.38	0.96	0.48	0.71

**Table 8:** Pearson correlation coefficients (*r*) amongst ten oenological measurements of white wine in twenty-one bentonite product treatments (Debrecen-Pallag, Hungary). Bold figures represent the significant (*p* < 0.05) correlation coefficient values among parameter pairs.

	Sugar	Acidity	Tartaric Acid	Malic Acid	Lactic Acid	Citric Acid	Volatile Acid	Sulphur Free	Sulphur Bound	Total Phenol
Alcohol	−0.104	0.050	**0.723**	−0.160	0.432	0.078	−0.101	0.247	−0.168	−0.058
Sugar		0.090	0.194	0.327	0.421	**0.707**	−**0.588**	0.047	0.114	−**0.824**
Acidity			0.311	**0.901**	−**0.624**	**0.556**	−**0.641**	0.032	**0.551**	−0.461
Tartaric acid				0.173	0.202	0.317	−0.331	0.127	0.013	−0.296
Malic acid					−0.549	**0.731**	−**0.737**	−0.011	**0.667**	−**0.631**
Lactic acid						0.126	−0.014	0.284	−0.306	−0.255
Citric acid							−**0.914**	0.297	**0.666**	−**0.953**
Volatile acid								−0.399	−**0.591**	**0.886**
Sulphur free									0.205	−0.281
Sulphur bound										−0.469

**Table 9:** Factor loadings calculated from Principal Axis Factor Analysis (Varimax rotation) for ten oenological measurements of white wine samples in twenty-one bentonite product treatments (Debrecen-Pallag, Hungary). Factor loadings above 0.69 were significant at *p* = 0.05. Bold figures represent the significant (*p* < 0.05) factor loadings.

Factors	1	2	3	4
Alcohol	0.0673	0.6123	**0.7638**	−0.0124
Sugar	**0.7031**	0.4352	−0.5239	−0.3815
Acidity	**0.7345**	−0.4723	0.4011	−0.1427
Tartaric acid	0.3422	0.4271	0.6021	−0.2572
Malic acid	**0.8517**	−0.4737	0.0931	−0.1582
Lactic acid	−0.0932	**0.9616**	−0.2218	0.1118
Citric acid	**0.9736**	0.1725	−0.1252	0.0731
Volatile acid	−**0.9444**	−0.1138	0.0048	−0.1352
Sulphur-free	0.2612	0.3132	0.0927	**0.6927**
Sulphur-bound	**0.7025**	−0.3529	−0.0136	0.4438
Total phenol	−**0.9138**	−0.3027	0.2361	0.0615
Variance	5.05	2.54	1.58	1.05
Explained variance (%)	45.4	22.7	14.4	9.5

**Table 10:** Pearson correlation coefficients (*r*) amongst nineteen chemical elements of white wine from Debrecen-Pallag, Hungary. Bold figures represent the significant (*p* < 0.05) correlation coefficient values among parameter pairs.

	B	Ca	K	Mg	Na	P	S	Cr	Mn	Fe	Co	Ni	Cu	Zn	Sr	Cd	Ba	Pb
Al	0.401	0.506	0.466	0.452	**0.853**	−0.74	0.439	**0.684**	0.506	**0.789**	0.508	0.381	−**0.588**	−0.051	**0.639**	0.473	**0.825**	**0.659**
B		**0.686**	**0.901**	**0.902**	0.435	**0.727**	0.499	**0.596**	0.315	**0.566**	**0.610**	**0.675**	−0.015	0.447	0.475	**0.549**	0.323	0.185
Ca			**0.645**	**0.632**	0.428	0.261	0.493	**0.569**	0.296	**0.570**	**0.583**	0.455	−0.514	0.135	0.514	**0.617**	0.320	0.311
K				**0.743**	0.452	0.456	0.461	0.539	0.354	0.503	0.438	0.540	−0.078	0.243	**0.594**	**0.716**	0.447	0.276
Mg					**0.592**	**0.765**	0.322	**0.728**	0.253	**0.679**	**0.772**	**0.799**	−0.131	0.531	0.395	0.433	0.459	0.241
Na						0.069	0.174	**0.838**	0.351	**0.889**	**0.742**	**0.662**	−0.518	0.090	0.418	0.468	**0.848**	**0.569**
P							0.173	0.215	−0.025	0.188	0.422	0.518	0.316	**0.628**	0.007	0.017	−0.011	−0.081
S								0.287	**0.591**	0.246	0.198	0.151	−0.091	0.161	**0.602**	0.451	0.059	0.232
Cr									0.369	**0.884**	**0.861**	**0.805**	−0.471	0.291	0.504	0.531	**0.632**	0.334
Mn										0.231	0.107	0.191	−0.258	0.153	**0.798**	0.369	0.334	0.329
Fe											**0.891**	**0.701**	−0.469	0.217	0.363	0.503	**0.693**	0.439
Co												**0.847**	−0.351	0.425	0.214	0.468	0.451	0.222
Ni													−0.191	0.537	0.362	**0.573**	0.472	0.201
Cu														0.121	−0.436	−0.321	−0.511	−0.438
Zn															0.131	0.120	0.053	−0.042
Sr																**0.681**	0.531	0.517
Cd																	0.453	0.427
Ba																		**0.761**

**Table 11:** Factor loadings from principal axis factor analysis (Varimax rotation) for nineteen chemical elements of white wine in twenty-one bentonite product treatments (Debrecen-Pallag, Hungary). Factor loadings above 0.69 were significant at *p* = 0.05. Bold figures represent the significant (*p* < 0.05) factor loadings.

Factors	1	2	3	4
B	**0.9741**	0.3656	0.5002	0.2957
Ca	**0.7816**	0.3864	0.5056	0.4579
K	**0.8444**	0.5144	0.4648	0.4279
Mg	**0.9252**	0.2729	0.5757	0.3572
P	**0.7453**	−0.0517	0.1829	−0.1635
S	0.5715	0.4071	0.1622	0.1337
Mn	0.2283	**0.8471**	0.3059	0.3751
Sr	0.4271	**0.9344**	0.3325	0.5392
Cd	0.2948	**0.7019**	0.329	0.3692
Zn	0.3211	0.0961	0.4952	−0.0346
Co	0.4717	0.2196	**0.9405**	0.5523
Ni	0.4526	0.3992	**0.8762**	0.4516
Fe	0.4303	0.3727	**0.8913**	**0.7501**
Cr	0.4253	0.4486	**0.8734**	**0.7054**
Na	0.3963	0.4183	**0.7832**	**0.9104**
Ba	0.2659	0.4598	0.5843	**0.9263**
Pb	0.179	0.4771	0.3355	**0.8211**
Al	0.3894	0.5109	0.6453	**0.9368**
Cu	−0.1203	−0.3699	−0.3207	−**0.7051**
Variance	9.28	3.11	2.08	1.02
Explained variance (%)	48.8	16.3	11.1	5.4

**Table 12:** Pearson correlation coefficients (*r*) amongst twenty-one volatile organic compounds (VOCs) of white wine from Debrecen-Pallag, Hungary. Bold figures represent the significant (*p* < 0.05) correlation coefficient values among parameter pairs. Ace: Acetic acid, Eth: Ethyl Acetate, 1-P2: 1-Propanol, 2-methyl, δ-Do: δ-Dodecalactone, Pro: Propanoic acid, ethyl ester, 1,3-D: 1,3-Dioxolane, 2,4,5-trimethyl-, 1-B3: 1-Butanol, 3-methyl-, 1-B2: 1-Butanol, 2-methyl-, (S)-, 4-Am: 4-Amino-1,5-pentandioic acid, Pr2: Propanoic acid, 2-methyl-, ethyl ester, Iso: Isobutyl acetate, Tri: Triethyl borate, But: Butanoic acid, ethyl ester, 1-He: 1-Hexanol, 1-Bu: 1-Butanol, 3-methyl-, acetate, Hex: Hexanoic acid, ethyl ester, Phl: Phenol, 2-methyl-, Phy: Phenol, 2-methoxy-, Pha: Phenylethyl Alcohol, Bud: Butanedioic acid, diethyl ester, Oct: Octanoic acid, ethyl ester.

	Ace	Eth	1-P2	δ-Do	Pro	1,3-D	1-B3	1-B2	4-Am	Pr2	Iso	Tri	But	1-He	1-Bu	Hex	Phl	Phy	Pha	Bud	Oct
1-Propanol	0.243	0.296	**0.777**	0.304	0.296	−0.012	0.458	0.540	0.062	0.241	**0.681**	0.343	0.267	0.338	0.278	0.425	0.342	0.205	0.261	0.463	0.324
Acetic acid		0.354	0.292	0.398	0.244	0.245	0.376	0.342	0.254	0.233	0.221	0.511	0.333	0.332	0.192	0.123	0.398	0.459	0.424	**0.666**	0.345
Ethyl Acetate			**0.942**	**0.759**	**0.911**	**0.841**	**0.928**	**0.905**	**0.643**	**0.912**	**0.617**	**0.595**	**0.981**	**0.921**	**0.924**	**0.849**	**0.641**	**0.551**	0.331	**0.575**	**0.921**
1-Propanol, 2-methyl-				**0.611**	**0.883**	**0.873**	**0.941**	**0.949**	**0.675**	**0.859**	**0.657**	**0.621**	**0.922**	**0.902**	**0.843**	**0.852**	**0.661**	**0.587**	0.352	**0.637**	**0.806**
δ-Dodecalactone					**0.705**	**0.638**	**0.757**	**0.736**	0.484	**0.651**	**0.592**	**0.538**	**0.723**	**0.748**	**0.664**	**0.682**	0.317	0.182	0.208	0.339	**0.823**
Propanoic acid, ethyl ester						**0.822**	**0.859**	**0.839**	**0.641**	**0.906**	**0.616**	0.493	**0.905**	**0.845**	**0.835**	**0.792**	0.541	0.454	0.337	**0.577**	**0.918**
1,3-Dioxolane, 2,4,5-trimethyl-							**0.741**	**0.675**	**0.791**	**0.861**	0.359	0.349	**0.817**	**0.833**	**0.759**	**0.626**	**0.571**	**0.614**	0.116	0.389	**0.755**
1-Butanol, 3-methyl-								**0.992**	**0.703**	**0.865**	**0.597**	**0.613**	**0.896**	**0.948**	**0.811**	**0.809**	**0.649**	0.532	0.349	**0.649**	**0.844**
1-Butanol, 2-methyl-, (S)-									**0.668**	**0.834**	**0.631**	**0.611**	**0.879**	**0.921**	**0.808**	**0.837**	**0.628**	0.486	0.358	**0.629**	**0.819**
4-Amino-1,5-pentandioic acid										**0.701**	0.148	0.358	**0.639**	**0.774**	**0.556**	**0.568**	**0.551**	**0.541**	0.178	0.404	0.478
Propanoic acid, 2-methyl-, ethyl ester											0.479	0.526	**0.903**	**0.891**	**0.881**	**0.781**	**0.589**	0.492	0.368	**0.601**	**0.863**
Isobutyl acetate												0.322	**0.594**	0.522	**0.641**	**0.591**	0.511	0.291	0.276	0.387	**0.669**
Triethyl borate													0.526	**0.633**	0.441	0.492	0.321	0.316	0.422	**0.672**	**0.601**
Butanoic acid, ethyl ester														**0.878**	**0.934**	**0.879**	**0.656**	0.536	0.416	0.549	**0.901**
1-Hexanol															**0.783**	**0.771**	**0.655**	**0.621**	0.338	**0.617**	**0.836**
1-Butanol, 3-methyl-, acetate																**0.858**	**0.577**	0.386	0.287	0.412	**0.836**
Hexanoic acid, ethyl ester																	0.391	0.246	0.322	0.382	**0.759**
Phenol, 2-methyl-																		**0.855**	0.474	**0.665**	0.515
Phenol, 2-methoxy-																			0.325	**0.636**	0.413
Phenylethyl Alcohol																				**0.616**	0.396
Butanedioic acid, diethyl ester																					**0.591**

**Table 13:** Factor loadings for twenty-two volatile organic compounds (VOCs) of white wine calculated from principal axis factor analysis (Varimax rotation). Factor loadings above 0.695 were significant at *p* = 0.05. Bold figures represent the significant (*p* < 0.05) factor loadings.

Factors	1	2	3	4
1-Propanol	**0.8137**	0.4664	0.5481	−0.0027
Ethyl Acetate	**0.9768**	**0.7093**	0.5431	0.0127
1-Propanol, 2-methyl-	**0.9466**	0.6876	0.5701	0.0255
Propanoic acid, ethyl ester	**0.9283**	0.6651	0.3996	0.0862
1,3-Dioxolane, 2,4,5-trimethyl-	**0.9312**	0.5819	0.4663	−0.0265
1-Butanol, 3-methyl-	**0.9495**	**0.7045**	0.5392	0.0243
1-Butanol, 2-methyl-, (S)-	**0.9589**	0.6916	0.5238	0.0128
Propanoic acid, 2-methyl-, ethyl ester	**0.9216**	0.6946	0.4557	0.0717
Isobutyl acetate	0.6708	0.331	0.3524	0.1087
Butanoic acid, ethyl ester	**0.9632**	**0.7051**	0.5197	0.0656
1-Hexanol	**0.9306**	**0.7186**	0.5856	0.0681
1-Butanol, 3-methyl-, acetate	**0.8566**	0.5236	0.4621	−0.0448
Hexanoic acid, ethyl ester	**0.8966**	0.5732	0.2664	−0.0381
Octanoic acid, ethyl ester	**0.905**	0.6682	0.3975	0.1221
2-Butanone	−0.4544	−**0.7175**	−0.3148	−0.1189
Triethyl borate	0.6447	**0.7943**	0.2695	0.2537
Phenylethyl Alcohol	0.3816	**0.7282**	0.4780	**0.7443**
Butanedioic acid, diethyl ester	0.6567	**0.8782**	0.6302	0.3334
4-Amino-1,5-pentandioic acid	0.6429	0.5318	0.3603	0.1211
Phenol, 2-methyl-	0.5615	0.5764	**0.9625**	0.2503
Phenol, 2-methoxy-	0.4206	0.4787	**0.9625**	0.2478
Acetic acid	0.0349	0.4583	0.2588	**0.9789**
Variance	14.8	2.11	1.23	0.99
Explained variance (%)	67.3	9.6	5.6	4.5

**Table 14:** Pearson correlation coefficients (*r*) amongst 10 organoleptic parameters of white wine from Debrecen-Pallag, Hungary. Bold figures represent the significant (*p* < 0.05) correlation coefficient values among parameter pairs.

	Colour	Flavor Intesity	Flavor Character	Flavor Quality	Taste Intensity	Taste Character	Taste Quality	Taste Persistency	Overall Harmony
Clearness	**0.829**	0.101	−0.276	−0.244	0.154	−0.215	0.147	0.036	−0.111
Colour		0.101	−0.159	−0.123	0.140	−0.280	0.245	−0.025	0.040
Flavor intensity			**0.612**	**0.573**	0.369	0.335	0.349	0.384	0.448
Flavor character				**0.671**	0.339	0.412	0.319	0.258	**0.581**
Flavor quality					**0.667**	0.457	0.432	0.510	**0.646**
Taste intensity						**0.594**	**0.571**	**0.815**	**0.701**
Taste character							0.339	**0.699**	0.469
Taste quality								0.528	**0.884**
Taste persistency									**0.654**

**Table 15:** Factor loadings for ten organoleptic parameters of white wine calculated from principal axis factor analysis (Varimax rotation). Factor loadings above 0.69 were significant at *p* = 0.05. Bold figures represent the significant (*p* < 0.05) factor loadings.

Factors	1	2	3	4
Clearness	0.0914	**0.9307**	0.0403	0.2415
Colour	0.0345	**0.9312**	0.1814	0.0128
Flavor intensity	−0.5537	0.0835	**0.7022**	0.3457
Flavor character	−0.5728	−0.3127	**0.7156**	0.0239
Flavor quality	−**0.8235**	−0.1911	0.2471	0.0562
Taste intensity	−**0.8487**	0.2339	−0.2979	0.1359
Taste character	−**0.7128**	−0.2563	−0.3774	0.3472
Taste quality	−**0.7371**	0.3428	−0.0659	−**0.6991**
Taste persistency	−**0.8175**	0.1148	−0.4518	0.2159
Overall harmony	−**0.8939**	0.0829	0.0112	−0.4292
Variance	4.82	2.21	1.19	0.83
Explained variance (%)	47.2	21.2	11.3	8.0

## Data Availability

Data is contained within the article.
